# Nutrients or nursing? Understanding how breast milk feeding affects child cognition

**DOI:** 10.1007/s00394-019-01929-2

**Published:** 2019-02-26

**Authors:** Wei Wei Pang, Pei Ting Tan, Shirong Cai, Doris Fok, Mei Chien Chua, Sock Bee Lim, Lynette P. Shek, Shiao-Yng Chan, Kok Hian Tan, Fabian Yap, Peter D. Gluckman, Keith M. Godfrey, Michael J. Meaney, Birit F. P. Broekman, Michael S. Kramer, Yap-Seng Chong, Anne Rifkin-Graboi

**Affiliations:** 1 Department of Obstetrics and Gynaecology, Yong Loo Lin School of Medicine, National University of Singapore, National University Health System, Singapore, 119228 Singapore; 2 Singapore Institute for Clinical Sciences (SICS), Agency for Science, Technology and Research (A*STAR), Singapore, 117609 Singapore; 3 Department of Neonatology, KK Women’s and Children’s Hospital, Singapore, 229899 Singapore; 4 Department of Child Development, KK Women’s and Children’s Hospital, Singapore, 229899 Singapore; 5 Department of Paediatrics, Yong Loo Lin School of Medicine, National University of Singapore, National University Health System, Singapore, 119228 Singapore; 6 Khoo Teck Puat-National University Children’s Medical Institute, National University Health System, Singapore, 119228 Singapore; 7 Department of Maternal Fetal Medicine, KK Women’s and Children’s Hospital, Singapore, 229899 Singapore; 8 Duke-NUS Medical School, Singapore, 169857 Singapore; 9 Department of Pediatric Endocrinology, KK Women’s and Children’s Hospital, Singapore, 229899 Singapore; 10 Liggins Institute, University of Auckland, 1142 Auckland, New Zealand; 11 Medical Research Council Lifecourse Epidemiology Unit, SO16 6YD Southampton, UK; 12 NIHR Southampton Biomedical Research Centre, University of Southampton and University Hospital Southampton NHS Foundation Trust, SO16 6YD Southampton, UK; 13 Departments of Psychiatry and Neurology, McGill University, Montreal, QC Canada; 14 Ludmer Centre for Neuroinformatics and Mental Health, Montreal, QC Canada; 15 Department of Psychiatry, VU Medical Centre, Amsterdam, The Netherlands; 16 Department of Pediatrics, Faculty of Medicine, McGill University, Montreal, QC H3A 1A2 Canada; 17 Department of Epidemiology, Biostatistics and Occupational Health, Faculty of Medicine, McGill University, Montreal, QC H3A 1A2 Canada; 18 National Institute of Education, 1 Nanyang Walk, Singapore, 637616 Singapore

**Keywords:** Breastfeeding, Breast milk expression, Child cognition, Memory

## Abstract

**Purpose:**

To explore the associations between type of milk feeding (the “nutrients”) and mode of breast milk feeding (the “nursing”) with child cognition.

**Methods:**

Healthy children from the GUSTO (Growing Up in Singapore Toward healthy Outcomes) cohort participated in repeated neurodevelopmental assessments between 6 and 54 months. For “nutrients”, we compared children exclusively bottle-fed according to type of milk received: formula only (*n* = 296) vs some/all breast milk (*n* = 73). For “nursing”, we included only children who were fully fed breast milk, comparing those fed directly at the breast (*n* = 59) vs those fed partially/completely by bottle (*n* = 63).

**Results:**

Compared to infants fed formula only, those who were bottle-fed breast milk demonstrated significantly better cognitive performance on both the Bayley Scales of Infant and Toddler Development (Third Edition) at 2 years [adjusted mean difference (95% CI) 1.36 (0.32, 2.40)], and on the Kaufman Brief Intelligence Test (Second Edition) at 4.5 years [7.59 (1.20, 13.99)]. Children bottle-fed breast milk also demonstrated better gross motor skills at 2 years than those fed formula [1.60 (0.09, 3.10)]. Among infants fully fed breast milk, those fed directly at the breast scored higher on several memory tasks compared to children bottle-fed breast milk, including the deferred imitation task at 6 months [0.67 (0.02, 1.32)] and relational binding tasks at 6 [0.41 (0.07, 0.74)], 41 [0.67 (0.04, 1.29)] and 54 [0.12 (0.01, 0.22)] months.

**Conclusions:**

Our findings suggest that nutrients in breast milk may improve general child cognition, while nursing infants directly at the breast may influence memory.

**Electronic supplementary material:**

The online version of this article (10.1007/s00394-019-01929-2) contains supplementary material, which is available to authorized users.

## Introduction

Though non-unanimous, numerous observational studies, meta-analyses, and randomized trial suggest breastfeeding improves child cognition [1–6]. Breastfeeding’s benefits appear greatest in studies of young children [6]. Several hypotheses may explain the association between breastfeeding and cognitive ability.

First, the benefits may be due to the nutritional contents of breast milk, like long-chain fatty acids such as docosahexaenoic acid (DHA) and arachidonic acid (AA), and their influence on brain development. DHA and AA together comprise approximately 20% of the brain’s fatty acid content and are involved in several aspects of early neurodevelopment, including modulation of cell growth and membrane lipid biosynthesis and myelination [7, 8]. Beyond fatty acids, breast milk also contains sialic acid, a key building block of brain ganglioside [9, 10], and other important nutrients for myelin synthesis, such as zinc, choline, and vitamin B_12_ [11]. Indeed, breastfeeding is linked to a faster rate of white matter development in brain regions associated with high-order cognition [12].

Second, breastfeeding might exert effects through the physical and/or emotional contact between mother and infant during breastfeeding [13, 14]. For example, greater maternal brain activation in response to breastfeeding has been associated with improved maternal sensitivity [15], which in turn is positively associated with infant language development [16]. Moreover, it is reasonable to think that direct breastfeeding associates with increased mother–child physical contact, and perhaps, skin-to-skin contact, which along with other forms of variation in exposure to maternal touch predict neurodevelopment [17].

Previous published studies on breastfeeding and child cognition have analyzed breastfeeding in terms of its duration and exclusivity. To our knowledge, these studies have not assessed whether associations with child cognition resulted from breast milk nutrients, the physical/emotional contact during breastfeeding, or a combination of both. Previous studies have not examined the relationship between breastfeeding mode—feeding directly at the breast vs feeding expressed breast milk (usually by bottle)—and child cognition, despite the increasing worldwide trend toward breast milk expression [18–20]. One randomized trial demonstrated a large benefit in cognition when preterm infants were tube fed breast milk vs infant formula, suggesting a positive effect of breast milk nutrients, but none of the infants received direct breastfeeding during hospitalization [21].

We previously reported significant associations between breastfeeding and child cognition among healthy, term infants in the first 2 years of life in the ‘Growing Up in Singapore Toward healthy Outcomes’ (GUSTO) study, comprised of multi-ethnic Asian Singaporeans [4]. We have also shown that breast milk expression is common, with a substantial fraction of GUSTO mothers feeding their infants expressed breast milk only instead of feeding directly at the breast [22]. Here, we use data from the same prospective cohort to explore the associations between mode of breast milk feeding (the “nursing”) and type of milk fed (the “nutrients”, i.e., breast milk vs formula) and child cognition, with a broad range of cognitive outcomes now extended to 4.5 years, and hypothesize that both “nursing” at the breast and the “nutrients” in breast milk feeding influence child cognitive ability.

## Methods

### Study design and population

In 2009 and 2010, women in their first trimester of pregnancy who were 18–46 years of age and of homogeneous (both parents) Chinese, Malay or Indian ethnicity were recruited from KK Women’s and Children’s Hospital (KKH) and National University Hospital (NUH) in Singapore into the GUSTO birth cohort study [23]. All children were offered a neurodevelopmental assessment at 48 months. Owing to limited availability of the evaluators, however, only a subset of children participated in the assessments conducted at 6, 18, 24, 41 and 54 months. The study was approved by the National Healthcare Group Domain Specific Review Board (NHG DSRB) and the Sing Health Centralised Institutional Review Board (CIRB). All participating mothers provided written informed consent.

**Fig. 1:**
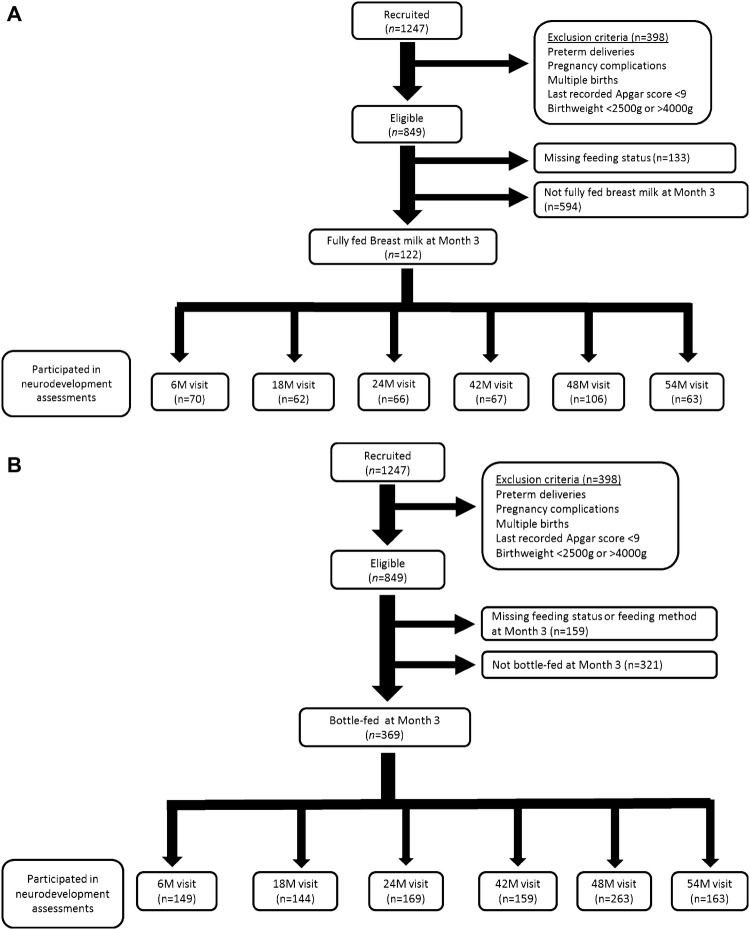
Flowchart of participants for analyses examining neurodevelopmental outcomes among different **a** types of nursing and **b** milk nutrients

Of 1247 mother–child dyads recruited, we excluded dyads from analyses if offspring were: not singletons; born preterm (< 37 weeks gestation); from pregnancies with complications (e.g., pre-eclampsia, gestational diabetes); with birth weight < 2500 g or > 4000 g; or had a last recorded Apgar score of < 9 at 5 or 10 min post-delivery (Fig. 1a, b).

For “nursing” analyses, comparing different modes of feeding breast milk, only children who were fully fed breast milk at 3 months postpartum were included (*n* = 122) (Fig. 1a). As detailed previously [22], fully breastfed included infants who were either exclusively breastfed (i.e., only received breast milk, including expressed breast milk) or those who were predominantly breastfed (i.e., received breast milk and may have received some non-milk liquids such as water and water-based drinks [including oral rehydration solution, fruit juices], or syrups and drops consisting of vitamins, minerals or medications). Very few children (2.5–3%) were predominantly breastfed in our cohort [22], with most of these predominantly breastfed infants receiving water, rather than other non-milk liquids. For “nutrient” analyses, comparing the consumption of breast milk vs formula, we included only children who were exclusively bottle-fed at 3 months postpartum (*n* = 369) (Fig. 1b).

The number of children with available neurocognitive data at each time point is indicated in Fig. 1. As some children had unusable data owing to fatigue, poor cooperation or fussiness, as well as technical errors (e.g., computer or video malfunction) particularly at 6 and 18 months, the number of children with usable data for each task differed.

### Data collection

Participants’ ethnic backgrounds, recruitment age and highest educational attainment were obtained from mothers at < 14 weeks gestation by trained research coordinators. Pregnancy complications (pre-eclampsia and gestational diabetes) and delivery details (gestational age, infant sex, Apgar scores, and birth weight) were extracted from medical records. Infants were classified into birth weight percentiles as described by Mikolajczyk et al. [24]. Mothers completed the State-Trait Anxiety Inventory (STAI) at 26–28 weeks’ gestation, as detailed previously in the GUSTO cohort [25].

Infant feeding type (exclusive breastfeeding, predominant breastfeeding, partial breastfeeding or formula only) and data pertaining to the age of breastfeeding cessation were ascertained at week 3, month 3 and every 3-month intervals thereafter until 12 months using interviewer-administered questionnaires. Any breast milk feeding refers to an infant receiving breast milk (either directly at the breast or fed expressed breast milk), with or without non-human milk and/or solids. At 3 months, breastfeeding mothers were asked how their infants were fed breast milk (at the breast, bottle only, and breast + bottle) [26]. Bottle only includes infants who received only breast milk expressed from the breast (either manually or via a pump) by bottle, cup or spoon (very few were fed by cup or spoon). Breast + bottle refers to infants fed directly at the breast but who also received some expressed breast milk by bottle (or cup or spoon).

Our primary outcome was child cognition assessed from 6 to 54 months. Neurocognitive assessments conducted at the different time points included paper and pencil/computerized tasks requiring motor and/or verbal responses, behavioral observation and eye tracking (Table 1). These assessments were conducted by personnel trained by GUSTO cohort investigators; for standardized tests like the Bayley Scales of Infant and Toddler Development, 3rd edition (BSID-III) and the Kaufman Brief Intelligence Test, 2nd edition (KBIT-2), personnel were trained by a psychologist/psychiatrist. With the exception of BSID-III and School Readiness Test which were conducted at participant’s home at 24 months and 48 months, respectively, all other neurocognitive assessments were performed at the clinic. The full details of the cognitive test methodologies are provided in the Supplementary Methods (Online Resource 1).

**Table 1:** Summary of neurocognitive assessments in 6–54-month-old children

Type of tasks	Time points					
	6 months	18 months	24 months	41 months	48 months	54 months
Memory	Habituation Deferred imitation Relational binding	Deferred imitation	Deferred imitation	Deferred imitation Relational Binding		Relational Binding
Executive functioning and self-regulation				Dimensional Card Sorting Task Snack & sticker delay		Dimensional Card Sorting Task
Attention/pre-attention and working Memory	Visual expectation	Visual expectation				CANTAB - spatial working memory
Social-emotional development						Novel word learning
Testing batteries			Bayley Scales of Infant Development III		School readiness test	Kaufman Brief Intelligence test - 2

Assessment details and references are shown in the Supplementary Methods (Online Resource 1)

### Statistical analyses

We conducted two separate analyses. In our “nursing” analyses, we analyzed breast milk feeding mode by including only children who were fully fed breast milk at 3 months. In this analysis, we compared those who were fed only directly at the breast; those fed directly at the breast who also received expressed breast milk (either manually or via a pump) by bottle, cup or spoon; and those who received only expressed breast milk. Since very few (*n* = 11) infants received expressed breast milk only, they were combined with the middle (direct + expressed) group (Supplemental Table 1, Online Resource 1).

In our “nutrient” analyses, we compared groups of children who were exclusively bottle-fed but who differed in the type of milk received: breast milk, formula, or a combination of both. Infants who were fed at the breast, either exclusively or partially, were excluded from the second analysis. Again, because very few (*n* = 11) infants were bottle-fed breast milk only, they were added to the combination group (Supplemental Table 1, Online Resource 1).

Cohort participants are described using proportions or means ± SD, with crude (unadjusted) comparisons of the types of nursing and milk nutrients based on Chi square tests or *t* tests. Adjusted associations of the types of nursing and milk nutrients with neurocognitive outcomes were examined using multivariable linear regression or logistic regression for continuous or dichotomous outcomes, respectively.

The choice of covariates included in multivariable models was based on our previous studies [27, 28]: ethnicity (Chinese, Malay, or Indian), maternal education (tertiary and non-tertiary), child’s sex, birth weight category [small for gestational age (SGA), appropriate for gestational age (AGA), and large for gestational age (LGA)], and antenatal maternal STAI-state scores. Participants (0–6%) with missing covariates were excluded from the statistical analyses. Sensitivity analyses using multiple imputation were also conducted; the results were similar and are, therefore, not presented. All statistical analyses were performed using SPSS version 24.0 (IBM Corp., Armonk, NY, USA).

## Results

### Participant characteristics

Among mothers who were feeding breast milk fully at 3 months, similar proportions fed their infants directly at the breast (48.4%) or partially/completely fed their infants breast milk by bottle (51.6%) (Table 2a). Girls and children of mothers without tertiary education, tended to be fed directly at the breast, rather than bottle-fed breast milk. Breast milk feeding duration was similar between the two groups. Among all mothers who bottle-fed their infants at 3 months, the majority of mothers fed their infants formula exclusively (80.2%), with 19.8% mothers feeding their infants some or all expressed breast milk (Table 2b). Mothers of Malay ethnicity, of younger age, without tertiary education or who were more anxious during pregnancy were more likely to bottle-feed their infants formula only. Not surprisingly, the duration of breast milk feeding was significantly longer among mothers who fed their infants some or all expressed breast milk when compared to those who fed their infants formula only at 3 months postpartum.

**Table 2:** Maternal and infant characteristics by (a) type of nursing and (b) milk nutrients at 3 months postpartum

Characteristics	(a) Nursing (breast milk only)				(b) Nutrients (fed by bottle)			
	All participants (*n* = 122)	At breast (*n* = 59, 48.4%)	Breast + bottle or bottle only (*n* = 63, 51.6%)	*p*	All participants (*n* = 369)	Formula only (*n* = 296, 80.2%)	Breast milk + formula or breast milk only (*n* = 73, 19.8%)	*p*
Ethnicity				0.057				< 0.001
Chinese	85	36 (42.4)	49 (57.6)		205	140 (68.3)	65 (31.7)	
Malay	16	8 (50.0)	8 (50.0)		108	106 (98.1)	2 (1.9)	
Indian	21	15 (71.4)	6 (28.6)		56	50 (89.3)	6 (10.7)	
Maternal age (year), mean ± SD	30.9 ± 4.4	31.0 ± 4.6	30.8 ± 4.2	0.814	29.4 ± 5.3	28.9 ± 5.4	31.6 ± 4.4	< 0.001
Maternal education^a^				0.001				< 0.001
Non-tertiary	39	27 (69.2)	12 (30.8)		281	256 (91.1)	25 (8.9)	
Tertiary	79	30 (38.0)	49 (62.0)		83	35 (42.2)	48 (57.8)	
Child’s sex				0.019				0.600
Male	63	24 (38.1)	39 (61.9)		187	148 (79.1)	39 (20.9)	
Female	59	35 (59.3)	24 (40.7)		182	148 (81.3)	34 (18.7)	
Child’s birth weight category				0.462				0.786
SGA (< 10%)	10	4 (40.0)	6 (60.0)		44	36 (81.8)	8 (18.2)	
AGA (10–90%)	94	44 (46.8)	50 (53.2)		278	224 (80.6)	54 (19.4)	
LGA (> 90%)	18	11 (61.1)	7 (38.9)		47	36 (76.6)	11 (23.4)	
STAI-state anxiety at 26 week pregnancy,^a^ mean ± SD	31.0 ± 8.7	31.6 ± 8.8	30.5 ± 8.5	0.504	35.4 ± 10.0	36.2 ± 10.1	31.9 ± 8.7	0.001
Duration of any breast milk feeding (month)^a^, mean ± SD	12.5 ± 3.3	12.7 ± 3.1	12.2 ± 3.5	0.405	2.0 ± 2.9	1.0 ± 0.8	7.3 ± 4.0	< 0.001

Data presented are *n* (%) unless otherwise stated

*AGA* appropriate for gestational age, *LGA* large for gestational age, *SGA* small for gestational age, *STAI* State-Trait Anxiety Inventory

^a^Number of participants with missing data: maternal education, (a) *n* = 4, and (b) *n* = 5; STAI-state Anxiety at 26 week pregnancy, (a) *n* = 4, and (b) *n* = 2; duration of any breast milk feeding, (a) *n* = 18, and (b) *n* = 43

## Nursing analyses

Significant differences in memory were observed among those fed directly at the breast vs those fed partially/completely by bottle. Specifically, for relational memory at 6 months, in the lag 2 trials, which encompassed both delay and interfering information, the proportion of time spent looking at the correctly matched picture in the third 1000-ms time bin was higher among those who received milk directly from the breast than among those fed partially/completely by bottle (*P* = 0.022) (Table 3a). No significant differences were observed by the type of nursing in the lag 0 trials, which involved neither delay nor interference from other stimuli (Table 3a). At 41 months, children fed directly at the breast were accurate in a higher proportion of trials than were those fed breast milk partially/completely by bottle in an aspect of the relational memory task that included face stimuli (*P* = 0.038). Children fed at the breast only also spent proportionally longer time looking at the correctly matched picture in the lag 2 trials conducted at 54 months (*P* = 0.031) (Table 3a).

**Table 3:** Associations between (a) the type of nursing and (b) milk nutrients with performance in relational binding

Relational binding (memory)		(a) Nursing				(b) Nutrients		
	N	Unadjusted mean ± SD		Adjusted mean differences (95% CI)^b, d^	N	Unadjusted mean ± SD		Adjusted mean differences (95% CI)^c, d^
		Breast + bottle or bottle only	At breast only	At breast only		Formula only	Breast milk + formula or breast milk only	Breast milk + formula or breast milk only
6 months								
Lag 0 trials (Time bins^a^)								
1000-ms Bin 1	34	0.31 ± 0.17	0.41 ± 0.21	0.13 (− 0.07, 0.33)	90	0.36 ± 0.22	0.23 ± 0.14	− 0.10 (− 0.23, 0.04)
1000-ms Bin 2	31	0.28 ± 0.19	0.32 ± 0.21	− 0.04 (− 0.20, 0.11)	85	0.32 ± 0.26	0.28 ± 0.21	− 0.02 (− 0.18, 0.13)
1000-ms Bin 3	29	0.30 ± 0.36	0.27 ± 0.28	− 0.08 (− 0.46, 0.31)	78	0.30 ± 0.32	0.24 ± 0.23	− 0.14 (− 0.35, 0.06)
Lag 2 trials (Time bins^a^)								
1000-ms Bin 1	33	0.43 ± 0.29	0.42 ± 0.31	− 0.07 (− 0.36, 0.21)	85	0.36 ± 0.23	0.43 ± 0.17	0.07 (− 0.07, 0.22)
1000-ms Bin 2	28	0.42 ± 0.17	0.40 ± 0.32	0.08 (− 0.19, 0.35)	81	0.35 ± 0.24	0.36 ± 0.29	0.06 (− 0.11, 0.24)
1000-ms Bin 3	24	0.29 ± 0.26	0.43 ± 0.30	0.41 (0.07, 0.74)^e^	70	0.46 ± 0.34	0.40 ± 0.33	0.04 (− 0.21, 0.29)
41 months								
Accuracy in food block	57	3.13 ± 1.07	2.84 ± 0.85	− 0.27 (− 0.86, 0.32)	109	2.89 ± 1.18	3.00 ± 1.06	0.25 (− 0.41, 0.91)
Accuracy in face block	57	2.35 ± 0.88	2.92 ± 1.09	0.67 (0.04, 1.29)^e^	108	2.56 ± 1.09	2.63 ± 0.77	0.30 (− 0.29, 0.89)
Combined food and face accuracy	58	5.41 ± 1.52	5.65 ± 1.44	0.45 (− 0.49, 1.39)	109	5.42 ± 1.67	5.63 ± 1.41	0.59 (− 0.33, 1.51)
Inference memory accuracy	56	1.23 ± 0.76	1.40 ± 0.91	0.29 (− 0.25, 0.84)	106	1.61 ± 0.87	1.25 ± 0.68	− 0.15 (− 0.64, 0.34)
54 months								
Lag 0 trials								
Accuracy	52	0.55 ± 0.29	0.67 ± 0.31	− 0.03 (− 0.26, 0.20)	115	0.55 ± 0.31	0.59 ± 0.26	0.02 (− 0.14, 0.19)
% Looking to correct match	52	0.37 ± 0.17	0.40 ± 0.16	− 0.09 (− 0.23, 0.04)	114	0.43 ± 0.21	0.45 ± 0.17	0.01 (− 0.11, 0.11)
Lag 2 trials								
Accuracy	52	0.41 ± 0.22	0.49 ± 0.32	0.08 (− 0.15, 0.32)	115	0.41 ± 0.27	0.53 ± 0.26	0.13 (− 0.02, 0.28)
% Looking to correct match	52	0.36 ± 0.15	0.39 ± 0.09	0.12 (0.01, 0.22)^e^	113	0.34 ± 0.13	0.42 ± 0.12	0.06 (− 0.01, 0.13)

^a^Time bins are defined in 1000-ms blocks after the pictures appear on the screen

^b^Values are adjusted mean differences (95% CI) from the reference group (Breast + bottle or bottle only)

^c^Values are adjusted mean differences (95% CI) from the reference group (Formula only)

^d^Values are adjusted for ethnicity (Chinese, Malay and Indian), maternal education (non-tertiary and tertiary), birth weight category (SGA, AGA, and LGA), 26-week STAI-state scores (continuous), child’s sex, and age during assessment (continuous)

^e^Values are *P* < 0.05 compared to the reference group

During the deferred imitation test, the number of target behaviors reproduced by 6-month-old infants was greater among those who were fed directly at the breast than among those bottle-fed breast milk (*P* = 0.043). Performance in other memory tasks, including habituation and deferred imitation at time points other than 6 months, was similar across the different types of nursing (Supplementary Table 2a, Online Resource 1).

Performance on testing batteries conducted at 24, 48 and 54 months are shown in Table 4 and Supplementary Table 3 (Online Resource 1). Among children “nursed” differently, a significant difference was observed for The Peabody Picture Vocabulary Test (PPVT) and for Weber Fraction, a part of Panamath; contrary to our hypothesis, children fed directly at the breast performed less well than those fed partially/completely by bottle, *P* = 0.039 and *P* = 0.013, respectively (Supplementary Table 3a, Online Resource 1). No other significant associations were observed. No significant associations were observed between type of nursing and tasks relating to executive functioning (dimensional card sorting tasks, sticker and snack delay), attention (visual expectation and CANTAB) or social-emotional development (novel word learning) (Supplementary Table 4a–6a, Online Resource 1).

**Table 4:** Associations between (a) the type of nursing and (b) milk nutrients with testing batteries

Testing batteries	(a) Nursing				(b) Nutrients			
	*N*	Unadjusted mean ± SD		Adjusted mean differences (95% CI)^a,c^	*N*	Unadjusted mean ± SD		Adjusted mean differences (95% CI)^b,c^
		Breast + bottle or bottle only	At breast only	At breast only		Formula only	Breast milk + formula or breast milk only	Breast milk + formula or breast milk only
BSID-III								
24 months								
Cognition	61	11.45 ± 2.56	10.29 ± 2.48	− 1.11 (− 2.55, 0.33)	157	9.62 ± 2.43	11.06 ± 2.24	1.36 (0.32, 2.40)^d^
Receptive language	61	10.15 ± 2.84	9.86 ± 2.03	− 0.21 (− 1.76, 1.33)	156	8.25 ± 2.58	9.65 ± 3.16	0.48 (− 0.70, 1.65)
Expressive language	61	9.97 ± 2.49	10.29 ± 2.77	− 0.06 (− 1.57, 1.46)	155	8.44 ± 2.15	9.58 ± 3.10	0.57 (− 0.47, 1.60)
Fine motor	61	10.45 ± 1.87	11.25 ± 2.27	1.08 (− 0.15, 2.31)	154	10.35 ± 2.27	11.17 ± 2.57	0.64 (− 0.56, 1.83)
Gross motor	61	11.91 ± 3.53	11.82 ± 2.75	− 0.33 (− 2.30, 1.64)	154	10.71 ± 3.00	12.17 ± 3.12	1.60 (0.09, 3.10)^d^
KBIT-2								
54 months								
Verbal	62	93.85 ± 13.32	95.62 ± 19.42	1.47 (− 9.53, 12.46)	158	79.87 ± 13.06	91.16 ± 17.15	6.50 (0.13, 12.87)^d^
Nonverbal	62	100.52 ± 15.99	106.17 ± 10.30	4.72 (− 4.30, 13.75)	159	95.71 ± 15.16	102.28 ± 13.32	6.28 (− 0.56, 13.11)
IQ	62	96.91 ± 13.95	101.34 ± 13.19	3.76 (− 5.75, 13.27)	158	86.10 ± 13.56	96.81 ± 14.03	7.59 (1.20, 13.99)^d^

*BSID-III* Bayley Scales of Infant and Toddler Development (Third Edition), *KBIT-2* Kaufman Brief Intelligence Test (Second Edition)

^a^Values are adjusted mean differences (95% CI) from the reference group (Breast + bottle or bottle only)

^b^Values are adjusted mean differences (95% CI) from the reference group (Formula only)

^c^Adjusted models include the covariates: ethnicity (Chinese, Malay, and Indian), maternal education (non-tertiary and tertiary), birth weight category (SGA, AGA, and LGA), 26-week STAI-state scores (continuous) and child’s sex

^d^Values are *P* < 0.05 compared to the reference group

## Nutrient analyses

Among all children who were bottle-fed during infancy, type of milk (breast milk vs formula) consumed was not significantly associated with performance in the memory tasks conducted at any follow-up time point (Table 4b, and Supplementary Table 2b, Online Resource 1).

Results showed an overall positive crude association between breast milk feeding and cognition domain scores, as well as gross motor scores, on the BSID-III (Table 4b). Even after adjusting for confounders, children who were fed some/only breast milk in the first 3 months had significantly higher cognition domain scores (*P* = 0.011), as well as gross motor scores than those who were fed only formula (*P* = 0.038). Children who were fed some/only breast milk also scored higher for the verbal component of the KBIT at 54 months than those who were fed formula only (*P* = 0.046); the overall score on the KBIT was also significantly higher (*P* = 0.020). No significant associations were observed between milk types and any of the school readiness tests at 48 months (Supplementary Table 3b, Online Resource 1).

Children who had been fed some/all breast milk had better use of strategy in the spatial working memory task than those fed formula only (*P* = 0.023) (Supplementary Table 5b, Online Resource 1). No significant associations were observed on tasks of executive functioning (dimensional card sorting, sticker and snack delay), attention (visual expectation) or social-emotional development (novel word learning) (Supplementary Tables 4–6, Online Resource 1). F-statistic and P values for the associations of the type of nursing or milk nutrients with cognitive assessments are shown in Supplementary Tables 7–11, Online Resource 1.

## Discussion

Our results suggest that contact accompanying feeding directly at the breast may contribute to brain development. This is consistent with prior, unexamined, hypotheses that the physical and emotional contact of direct breastfeeding (the nursing), in addition to the nutritional content of breast milk may confer benefits in child cognition. Here, we observed that whilst breast milk can improve the child’s general cognition, motor skills, as well as language abilities, direct breastfeeding appears to influence their memory.

Compared to children fed infant formula only during early infancy, those fed expressed breast milk demonstrated significantly better cognitive performance at 2 and 4.5 years, even after adjusting for maternal education, age and anxiety level during pregnancy. Higher IQ scores at 4.5 years appear to be driven by improved verbal skills; the association between breast milk intake and higher scores on nonverbal tasks was of only borderline statistical significance. We observed no significant differences in 2-year-old language tasks, nor on any of the 4-year-old school readiness tests, although the mean scores for those who consumed breast milk were generally higher. Results of previous breastfeeding and cognition studies are not directly comparable to ours, because in past work “breastfeeding” refers to infants fed directly at the breast and/or fed expressed breast milk. Nevertheless, many studies have reported better cognitive performance [3, 29, 30] and language abilities [3, 5, 30] among children who had consumed more breast milk as infants. Various milk nutrients have been hypothesized to contribute to improved child cognitive ability, including long-chain polyunsaturated fatty acids, such as AA and DHA [31–33] (which are important for cognitive maturation [34]). Nonetheless, randomized trials of feeding formula supplemented with these nutrients have not confirmed those hypotheses [35].

GUSTO children fed expressed breast milk also demonstrated better gross motor skills at age 2 years than those fed formula only. Previous studies of motor skills in relation to breastfeeding have reported inconsistent results [1, 12, 30, 36, 37]. Even among studies that conducted the same motor tests (i.e., BSID) at approximately 2 years of age have reported mixed results [30, 36]. One explanation for this disparity is that past work did not examine both nutritional and nursing influences on motor development. Further studies with larger samples are needed.

Among GUSTO children who were exclusively fed breast milk, those fed directly at the breast scored higher on several memory tasks compared to children fed breast milk via bottles. In particular, they reproduced more target actions during the deferred imitation task at 6 months and showed evidence of better relational binding at 6, 41 and 54 months of age. Deferred imitation requires a child to reproduce previously learned actions and so indicates recollection of past events. The relational binding task requires children to bind together different aspects of an experience, scene, etc., and is important to autobiographical memory and learning [17]. Both deferred imitation [38] and relational binding [39, 40] may reflect memory processes that primarily involve the hippocampus, a region of the brain essential for flexible memory expression [41].

How the act of breastfeeding benefits memory is unknown. The benefits are unlikely due to differences in the feeding frequencies, as the nutrients that contribute to infant satiety, and, therefore, to feeding frequency, are nearly identical for both modes of breast milk feeding. The benefits to memory may be due to differences in the frequency and/or duration of mother–infant contact. For example, direct skin-to-skin contact, perhaps more likely in children fed at the breast, may influence a variety of processes including pain sensitivity and stress responsivity. Variation in stress may be especially influential to memory processes. Many studies have reported that exposure to stress or an elevated level of corticosteroids alters performance on memory tasks that are dependent on the hippocampus [42, 43]. In animal studies, stress alters ensuing synaptic plasticity and firing properties of hippocampal neurons. Additionally, both human and animal studies have shown that stress can change neuronal morphology, suppress neuronal proliferation, alter hippocampal volume [44, 45], and, perhaps alter the time course of hippocampal growth [46]. Varying levels of hypothalamic–pituitary–adrenal axis neuroendocrine hormones, particularly glucocorticoids, appear to mediate the myriad stress effects on the hippocampus [45].

Our study’s strengths include assessment of numerous specific cognitive measures, as well as the use of generalized cognitive test batteries. Moreover, cognitive measures were obtained at several time points from early infancy to 4.5 years. We were also able to control for a large number of potential confounding factors. One study limitation is our definition of the type of nursing, which was defined at 3 months of age. As a result, we were unable to examine whether the neurocognitive outcomes would be similar if the type of nursing was also compared at later ages. However, of the mothers who continued to breastfeed to 6 months (< 50% of the cohort), the majority (> 70%) maintained the same type of nursing at 3 and 6 months, suggesting that nursing type at 3 months is a valid surrogate of longer term feeding. We also have modest statistical power for some analyses, owing to small sample sizes for some cognitive measures conducted. Finally, we examined many cognitive outcomes, most of the associations we observed were of modest magnitude, and some were opposite in direction to our hypothesis. Some of our results may, therefore, reflect the play of chance.

Nevertheless, ours is the first study that has attempted to disentangle the potential effects on child cognitive ability of the nutrients in breast milk vs the act of nursing implicit in direct breastfeeding.

Our results suggest that breastfeeding’s impact on brain development may be due to both factors. Although the significant associations we observed were modest in magnitude and limited to some tests at specific ages, our findings suggest that the nutritional content of breast milk may improve general child cognition, language abilities and gross motor skills, while feeding infants directly at the breast may influence memory abilities. Such work may be of direct relevance to maternal child postpartum well-being and pediatric practice: anecdotally, mothers often interpret advice to breastfeed as advice to provide breast milk, and pumping breast milk may be a preferred means of administration in some cultures. As breast-pump technology becomes increasingly advanced and accessible, providing breast milk may become further removed from at-the-breast feeding. Future studies with larger sample sizes and higher exclusive breastfeeding rates will be important to confirm or refute our findings.

## Electronic supplementary material

Below is the link to the electronic supplementary material.

Supplementary material 1 (DOCX 123 KB)
