# Supplementary material for: Nutrients or nursing? Understanding how breast milk feeding affects child cognition
Source: Eur J Nutr. 2019 Feb 26;59(2):609–19. doi: 10.1007/s00394-019-01929-2 (PMC7058674; doi:10.1007/s00394-019-01929-2)
Supplement: Supplementary file 1 — Supplementary material 1 (DOCX 123 KB) [file 394_2019_1929_MOESM1_ESM.docx]

**Title:** Nutrients or nursing? Understanding how breast milk feeding affects child cognition

**Journal:** European Journal of Nutrition

**Author:** Wei Wei Pang, Pei Ting Tan, Shirong Cai, Doris Fok, Mei Chien Chua, Sock Bee Lim, Lynette P Shek, Shiao-Yng Chan, Kok Hian Tan, Fabian Yap, Peter D Gluckman, Keith M Godfrey, Michael J Meaney, Birit FP Broekman, Michael S Kramer, Yap-Seng Chong, Anne Rifkin-Graboi.

**Corresponding author:**

Anne Rifkin-Graboi

National Institute of Education, Singapore 637616, Singapore.

Email address: anne.rifkin@nie.edu.sg

**Electronic Supplementary material included in this file:**

Supplementary Table 1. Distribution of Children by the type of nursing and milk nutrients.

Supplementary Table 2. Associations between (a) the type of nursing and (b) milk nutrients with performance in memory assessments.

Supplementary Table 3. Associations between (a) the type of nursing and (b) milk nutrients with testing batteries.

Supplementary Table 4. Associations between (a) the type of nursing and (b) milk nutrients with assessments of executive functioning and self-regulation.

Supplementary Table 5. Associations between (a) the type of nursing and (b) milk nutrients with performance in attention/pre-attention and working memory assessments.

Supplementary Table 6. Association between (a) the type of nursing and (b) milk nutrients and social emotional development assessments.

Supplementary Table 7. F-statistic and P-values for the association between (a) the type of nursing and (b) milk nutrients with performance in memory assessments.

Supplementary Table 8. F-statistic and P-values for the association between (a) the type of nursing and (b) milk nutrients with performance in testing batteries.

Supplementary Table 9. F-statistic and P-values for the association between (a) the type of nursing and (b) milk nutrients with assessments of executive functioning and self-regulation.

Supplementary Table 10. F-statistic and P-values for the association between (a) the type of nursing and (b) milk nutrients with assessments of attention/ pre-attention and working memory.

Supplementary Table 11. F-statistic and P-values for the association between (a) the type of nursing and (b) milk nutrients with social emotional development assessments.

Supplementary Methods: Detailed descriptions of the cognitive test methodologies from 6 to 54 months.

**Supplementary Table 1: Distribution of Children by the type of nursing and milk nutrients**

|  | **Nursing Sample (Breast milk only), *n* = 122** | | |
| --- | --- | --- | --- |
|  | Bottle only | Breast + bottle | At breast |
| Frequency | 11 | 52 | 59 |
| Used in Analysis | 63 | | 59 |
|  | **Nutrients Sample (Fed by bottle), *n* = 369** | | |
|  | Breast milk only | Breast milk + Formula | Formula only |
| Frequency | 11 | 62 | 296 |
| Used in Analysis | 73 | | 296 |

**Supplementary Table 2. Associations between (a) the type of nursing and (b) milk nutrients with performance in memory assessments.**

|  |  | (a) Nursing | | | | | (b) Nutrients | | | | | | | | |  |
| --- | --- | --- | --- | --- | --- | --- | --- | --- | --- | --- | --- | --- | --- | --- | --- | --- |
|  | *N* | Unadjusted mean ± SD | |  | Adjusted mean differences (95% CI)^1,3^ |  | | | Unadjusted mean ± SD | | | |  | | Adjusted mean differences (95% CI)^2,3^ | |
| Assessments of memory |  | Breast + bottle or bottle only | At breast only |  | At breast only |  | | *N* | | Formula only | Breast milk + formula or breast milk only |  | | Breast milk + formula or breast milk only | |  |
| Habituation |  |  |  |  |  |  | |  | |  |  |  | |  | |  |
| 6 months |  |  |  |  |  |  | |  | |  |  |  | |  | |  |
| Proportion of time looking at novel stimuli | 42 | 0.51 ± 0.13 | 0.55 ± 0.14 |  | 0.03 (-0.09, 0.14) |  | | 102 | | 0.55 ± 0.12 | 0.48 ± 0.16 |  | | -0.08 (-0.15, 0.0002) | |  |
| Number of habituation trials | 37 | 9.95 ± 5.14 | 9.87 ± 4.73 |  | -1.76 (-6.77, 3.23) |  | | 85 | | 10.38 ± 4.78 | 9.55 ± 5.10 |  | | 0.29 (-2.57, 3.15) | |  |
|  |  |  |  |  |  |  | |  | |  |  |  | |  | |  |
| Deferred imitation |  |  |  |  |  |  | |  | |  |  |  | |  | |  |
| 6 months |  |  |  |  |  |  | |  | |  |  |  | |  | |  |
| Absolute score | 45 | 0.93 ± 0.78 | 1.11 ± 0.96 |  | 0.67 (0.02, 1.32)^4^ |  | | 83 | | 0.92 ± 0.91 | 1.17 ± 0.79 |  | | 0.24 (-0.31, 0.79) | |  |
|  |  |  |  |  |  |  | |  | |  |  |  | |  | |  |
| 18 months |  |  |  |  |  |  | |  | |  |  |  | |  | |  |
| Rattle task |  |  |  |  |  |  | |  | |  |  |  | |  | |  |
| Absolute score | 19 | 1.54 ± 1.05 | 2.00 ± 0.63 |  | 0.99 (-1.19, 3.18) |  | | 40 | | 1.68 ± 1.04 | 1.67 ± 1.37 |  | | 1.31 (-0.37, 3.00) | |  |
| Sequential score | 43 | 0.91 ± 0.79 | 0.90 ± 0.79 |  | -0.05 (-0.83, 0.72) |  | | 87 | | 0.85 ± 0.74 | 0.95 ± 0.78 |  | | 0.50 (-0.12, 1.11) | |  |
|  |  |  |  |  |  |  | |  | |  |  |  | |  | |  |
| 24 months |  |  |  |  |  |  | |  | |  |  |  | |  | |  |
| Absolute score | 54 | 0.96 ± 0.88 | 1.27 ± 1.15 |  | 0.08 (-0.60, 0.76) |  | | 127 | | 1.12 ± 1.02 | 1.38 ± 0.90 |  | | 0.17 (-0.35, 0.68) | |  |
| Sequential score | 56 | 0.19 ± 0.40 | 0.52 ± 0.82 |  | 0.25 (-0.17, 0.67) |  | | 133 | | 0.17 ± 0.46 | 0.44 ± 0.77 |  | | 0.27 (-0.02, 0.55) | |  |
|  |  |  |  |  |  |  | |  | |  |  |  | |  | |  |
|  |  |  |  |  |  |  | |  | |  |  |  | |  | |  |
|  |  |  |  |  |  |  | |  | |  |  |  | |  | |  |
|  |  |  |  |  |  |  | |  | |  |  |  | |  | |  |
|  |  |  |  |  |  |  | |  | |  |  |  | |  | |  |
|  |  | (a) Nursing | | | | | (b) Nutrients | | | | | | | | |  |
|  | *N* | Unadjusted mean ± SD | |  | Adjusted mean differences (95% CI)^1,3^ |  | | | Unadjusted mean ± SD | | | |  | | Adjusted mean differences (95% CI)^2,3^ | |
| Assessments of memory |  | Breast + bottle or bottle only | At breast only |  | At breast only |  | | *N* | | Formula only | Breast milk + formula or breast milk only |  | | Breast milk + formula or breast milk only | |  |
| 41 months |  |  |  |  |  |  | |  | |  |  |  | |  | |  |
| Absolute score for Dragonfly task | 55 | 4.10 ± 1.52 | 3.81 ± 1.74 |  | -0.29 (-1.28, 0.71) |  | | 120 | | 3.40 ± 1.65 | 3.65 ± 1.37 |  | | 0.46 (-0.45, 1.36) | |  |
| Absolute score for Giraffe task | 57 | 2.13 ± 1.68 | 2.11 ± 1.85 |  | 0.15 (-0.99, 1.28) |  | | 116 | | 1.80 ± 1.43 | 2.05 ± 1.46 |  | | -0.14 (-1.01, 0.74) | |  |
| Absolute score for Bird task | 55 | 3.03 ± 1.54 | 2.16 ± 1.77 |  | -0.99 (-2.10, 0.12) |  | | 117 | | 2.86 ± 1.53 | 2.50 ± 1.44 |  | | -0.12 (-1.04, 0.81) | |  |

^1^ Values are adjusted mean differences (95% CI) from the reference group (Breast + bottle or bottle only).

^2^ Values are adjusted mean differences (95% CI) from the reference group (Formula only).

^3^ Values are adjusted for ethnicity (Chinese, Malay Indian), maternal education (non-tertiary, tertiary), birth weight category (SGA, AGA, LGA), 26-wk STAI-state scores (continuous), child’s sex, age during assessment (continuous).

^4^ Values are P<0.05 compared to the reference group.

**Supplementary Table 3. Associations between (a) the type of nursing and (b) milk nutrients with testing batteries.**

|  |  | (a) Nursing | | | |  | | (b) Nutrients | | | | | | | | |  |  |
| --- | --- | --- | --- | --- | --- | --- | --- | --- | --- | --- | --- | --- | --- | --- | --- | --- | --- | --- |
|  | *N* | Unadjusted mean ± SD | |  | Adjusted mean differences (95% CI)^1^ |  |  | | | Unadjusted mean ± SD | |  | | | Adjusted mean differences (95% CI)^2^ | | | |
| Testing batteries |  | Breast + bottle or bottle only | At breast only |  | At breast only |  | *N* | | Formula only | | Breast milk + formula or breast milk only | |  | | Breast milk + formula or breast milk only | | |  |
| School readiness test | |  |  |  |  |  |  | |  | |  |  | |  | |  |  |  |
| 48 months |  |  |  |  |  |  |  | |  | |  |  | |  | |  |  |  |
| Lollipop score^3^ | 104 | 48.98 ± 8.88 | 45.04 ± 10.87 |  | -4.40 (-9.01, 0.21) |  | 253 | | 37.34 ± 14.76 | | 46.04 ± 12.07 |  | | 1.01 (-3.80, 5.81) | |  |  |  |
| NK test scores^3^ |  |  |  |  |  |  |  | |  | |  |  | |  | |  |  |  |
| Test 0 | 103 | 4.26 ± 0.77 | 3.91 ± 1.17 |  | -0.33 (-0.80, 0.15) |  | 257 | | 3.33 ± 1.49 | | 3.98 ± 1.35 |  | | 0.21 (-0.31, 0.72) | |  |  |  |
| Test 1 | 103 | 3.16 ± 3.41 | 2.76 ± 3.62 |  | -0.33 (-2.10, 1.45) |  | 257 | | 1.49 ± 2.47 | | 2.75 ± 3.17 |  | | 0.07 (-0.86, 0.99) | |  |  |  |
| Total | 103 | 7.42 ± 3.75 | 6.67 ± 4.15 |  | -0.65 (-2.62, 1.32) |  | 257 | | 4.82 ± 3.36 | | 6.73 ± 3.90 |  | | 0.27 (-0.94, 1.49) | |  |  |  |
| PPVT score^3^ | 104 | 98.57 ± 16.29 | 90.79 ± 16.89 |  | -8.46 (-16.49, -0.44)^5^ |  | 258 | | 82.25 ± 16.44 | | 92.86 ± 17.67 |  | | 4.01 (-1.72, 9.73) | |  |  |  |
| ROST score^3^ | 102 | 5.07 ± 2.59 | 4.30 ± 2.61 |  | -0.67 (-1.98, 0.64) |  | 257 | | 4.95 ± 3.11 | | 4.93 ± 3.00 |  | | -0.001 (-1.12, 1.12) | |  |  |  |
| VCR score^3^ | 95 | 2.48 ± 1.13 | 2.30 ± 1.28 |  | -0.44 (-1.04, 0.16) |  | 244 | | 2.05 ± 1.54 | | 2.15 ± 1.38 |  | | -0.18 (-0.74, 0.38) | |  |  |  |
| CTOPP2 scores^4^ |  |  |  |  |  |  |  | |  | |  |  | |  | |  |  |  |
| Elision | 103 | 9.18 ± 2.29 | 8.80 ± 1.92 |  | 0.11 (-0.96, 1.18) |  | 245 | | 8.18 ± 0.96 | | 8.55 ± 1.57 |  | | 0.12 (-0.30, 0.54) | |  |  |  |
| Blending Words | 101 | 9.13 ± 1.81 | 8.84 ± 1.72 |  | -0.01 (-0.90, 0.89) |  | 243 | | 8.31 ± 1.40 | | 8.78 ± 1.30 |  | | 0.01 (-0.51, 0.53) | |  |  |  |
| Sound matching | 103 | 10.81 ± 2.31 | 10.48 ± 2.34 |  | -0.49 (-1.59, 0.62) |  | 244 | | 9.87 ± 2.04 | | 10.49 ± 2.52 |  | | -0.30 (-1.09, 0.48) | |  |  |  |
| Phonological awareness | 100 | 98.52 ± 10.75 | 96.84 ± 8.73 |  | 0.09 (-5.10, 5.28) |  | 239 | | 92.87 ± 6.49 | | 95.56 ± 8.06 |  | | -0.65 (-3.14, 1.85) | |  |  |  |
| Panamath^3^ |  |  |  |  |  |  |  | |  | |  |  | |  | |  |  |  |
| % correct trials | 48 | 78.13 ± 8.84 | 77.94 ± 10.14 |  | -6.36 (-12.88, 0.17) |  | 73 | | 75.10 ± 9.67 | | 75.38 ± 7.42 |  | | -0.92 (-6.07, 4.23) | |  |  |  |
| Weber fraction | 48 | 0.54 ± 0.28 | 0.58 ± 0.39 |  | 0.28 (0.06, 0.50)^5^ |  | 73 | | 0.67 ± 0.37 | | 0.61 ± 0.25 |  | | -0.03 (-0.22, 0.17) | |  |  |  |

*NK* Number Knowledge, *PPVT* Peabody Picture Vocabulary Test, *ROST* Random Object Span Test, *VCR* Visually Cued Recall Test, *CTOPP2* Comprehensive Test of Phonological Processing-2.

^1^Values are adjusted mean differences (95% CI) from the reference group (Breast + bottle or bottle only).

^2^Values are adjusted mean differences (95% CI) from the reference group (Formula only).

^3^Adjusted models include the covariates: ethnicity (Chinese, Malay Indian), maternal education (non-tertiary, tertiary), birth weight category (SGA, AGA, LGA), 26-wk STAI-state scores (continuous), child’s sex and age at testing.

^4^Adjusted models include the covariates: ethnicity (Chinese, Malay Indian), maternal education (non-tertiary, tertiary), birth weight category (SGA, AGA, LGA), 26-wk STAI-state scores (continuous) and child’s sex.

^5^Values are P<0.05 compared to the reference group.

**Supplementary Table 4. Associations between (a) the type of nursing and (b) milk nutrients with assessments of executive functioning and self-regulation.**

|  |  | (a) Nursing | | | | | (b) Nutrients | | | | | | | | | | | | | | | | | | | | |
| --- | --- | --- | --- | --- | --- | --- | --- | --- | --- | --- | --- | --- | --- | --- | --- | --- | --- | --- | --- | --- | --- | --- | --- | --- | --- | --- | --- |
| Assessments of executive functioning and self-regulation | *N* | Unadjusted OR (95% CI) | |  | Adjusted OR (95% CI)^1,3^ |  | | |  | | | | | | | Unadjusted OR (95% CI) | | | |  | | | | Adjusted OR (95% CI)^2,3^ | | | |
|  |  | Breast + bottle or bottle only | At breast only |  | At breast only | | |  | | | *N* | | | Formula only | | | | Breast milk + formula or breast milk only | | |  | | | | Breast milk + formula or breast milk only | | |
| Dimensional change card sorting task | | |  |  |  |  | | | | | |  | | | |  | | |  | |  | | | |  | | |
| 41 months |  |  |  |  |  |  | | | | | |  | | | |  | | |  | |  | | | |  | | |
| Passed Emotion task | 47 | reference | 0.86 (0.24, 3.09) |  | 0.39 (0.01, 15.92) |  | | | | 84 | | | reference | | | | 3.69 (1.19, 11.41) | | |  | | | 0.49 (0.05, 4.97) | | | |  |
| Passed Food task | 50 | reference | 0.91 (0.26, 3.21) |  | 2.43 (0.32, 18.35) |  | | | | 86 | | | reference | | | | 2.45 (0.75, 7.96) | | |  | | | 0.91 (0.15, 5.69) | | | |  |
|  |  |  |  |  |  |  | | | |  | | |  | | | |  | | |  | | |  | | | |  |
| 54 months |  |  |  |  |  |  | | | |  | | |  | | | |  | | |  | | |  | | | |  |
| Performance in emotion task | 47 | reference | 2.23 (0.69, 7.21) |  | 3.16 (0.50, 20.05) |  | | | | 101 | | | reference | | | | 1.09 (0.41, 2.88) | | |  | | | 0.89 (0.24, 3.26) | | | |  |
| Performance in orientation task | 45 | reference | 1.83 (0.53, 6.34) |  | 1.21 (0.14, 10.70) |  | | | | 103 | | | reference | | | | 1.15 (0.43, 3.11) | | |  | | | 1.54 (0.41, 5.78) | | | |  |
|  |  |  |  |  |  |  | | | | | |  | | |  | | | |  | | |  | | | |  | |
|  |  | **(a) Nursing** | | | | **(b) Nutrients** | | | | | | | | | | | | | | | | | | | | | |
|  | *N* | Unadjusted mean ± SD | |  | Adjusted mean differences (95% CI)^3,4^ |  | | |  | | | | | | | Unadjusted mean ± SD | | | |  | | | | Adjusted mean differences (95% CI)^3,5^ | | | |
| Assessments of executive functioning and self-regulation |  | Breast + bottle and bottle only | At breast only |  | At breast only |  | | | | | | *N* | | | | Formula only | | | Breast milk + formula and breast milk only | |  | | | | Breast milk + formula and breast milk only | | |
| Sticker and Snack delay |  |  |  |  |  |  | | | | | |  | | | |  | | |  | |  | | | |  | | |
| 41 months |  |  |  |  |  |  | | | | | |  | | | |  | | |  | |  | | | |  | | |
| Snack Delay score | 60 | 8.16 ± 0.91 | 8.01 ± 1.07 |  | -0.20 (-0.82, 0.43) |  | | | | | | 126 | | | | 7.76 ± 1.45 | | | 8.01 ± 1.02 | |  | | | | 0.28 (-0.45, 1.00) | | |
| Sticker Delay score | 61 | 7.97 ± 0.88 | 7.56 ± 1.56 |  | -0.70 (-1.49, 0.10) |  | | | | | | 124 | | | | 7.87 ± 1.02 | | | 7.67 ± 1.73 | |  | | | | -0.08 (-0.74, 0.57) | | |
| Snack & Sticker Delay score | 61 | 8.14 ± 0.69 | 7.81 ± 1.19 |  | -0.61 (-1.23, 0.20) |  | | | | | | 112 | | | | 7.92 ± 0.98 | | | 7.89 ± 1.31 | |  | | | | 0.10 (-0.50, 0.71) | | |

^1^Values are adjusted odds ratio (95% CI) from the reference group (Breast + bottle or bottle only).

^2^Values are adjusted odds ratio (95% CI) from the reference group (Formula only).

^3^Adjusted models include the covariates: ethnicity (Chinese, Malay Indian), maternal education (non-tertiary, tertiary), birth weight category (SGA, AGA, LGA), 26-wk STAI-state scores (continuous), child’s sex and age at testing.

^4^Values are adjusted mean differences (95% CI) from the reference group (Breast + bottle or bottle only).

^5^Values are adjusted mean differences (95% CI) from the reference group (Formula only).

**Supplementary Table 5. Associations between (a) the type of nursing and (b) milk nutrients with performance in attention/pre-attention and working memory assessments.**

| Assessments of attention/ pre-attention and working memory |  | (a) Nursing | | | | |  | | (b) Nutrients | | | | | | | | | |
| --- | --- | --- | --- | --- | --- | --- | --- | --- | --- | --- | --- | --- | --- | --- | --- | --- | --- | --- |
|  | *N* | Unadjusted mean ± SD | |  | Adjusted mean differences (95% CI)^1,3^ | |  | | | Unadjusted mean ± SD | | | | |  | | | Adjusted mean differences (95% CI)^2,3^ |
|  |  | Breast + bottle or bottle only | At breast only |  | At breast only |  | | *N* | | Formula only | | | Breast milk + formula or breast milk only | | |  | Breast milk + formula or breast milk only | |
| Visual expectation |  |  |  |  |  | | |  | | | |  | |  | |  |  | |
| 6 months |  |  |  |  |  | | |  | | | |  | |  | |  |  | |
| Reaction time at experimental phase (ms) | 29 | 336.03 ± 54.45 | 329.00 ± 31.62 |  | -4.59 (-45.77, 36.58) | | | 88 | | | 322.50 ± 39.15 | | | 308.98 ± 29.95 | |  | -12.17 (-37.77, 13.42) | |
| Proportion of time looking at correct location (%) | 32 | 2.16 ± 2.53 | 2.10 ± 2.48 |  | 0.18 (-2.48, 2.84) | | | 89 | | | 2.52 ± 2.98 | | | 1.92 ± 3.55 | |  | -0.97 (-2.99, 1.04) | |
|  |  |  |  |  |  | | |  | | |  | | |  | |  |  | |
| 18 months |  |  |  |  |  | | |  | | |  | | |  | |  |  | |
| Reaction time at experimental phase (ms) | 39 | 597.94 ± 138.70 | 566.95 ± 117.42 |  | -27.78 (-132.41, 76.85) | | | 90 | | | 566.99 ± 101.08 | | | 590.09 ± 163.02 | |  | 17.85 (-59.57, 95.26) | |
| Proportion of time looking at correct location (%) | 39 | 4.73 ± 4.75 | 3.08 ± 4.43 |  | -2.14 (-5.42, 1.14) | | | 91 | | | 3.51 ± 2.71 | | | 3.94 ± 2.19 | |  | 0.29 (-1.37, 1.96) | |
|  |  |  |  |  |  | | |  | | |  | | |  | |  |  | |
| CANTAB Spatial working memory |  |  |  |  |  | | |  | | |  | | |  | |  |  | |
| 54 months |  |  |  |  |  | | |  | | |  | | |  | |  |  | |
| SWM Between errors | 58 | 72.41 ± 12.38 | 74.23 ± 15.72 |  | 1.04 (-8.37, 10.45) | | | 153 | | | 75.02 ± 14.54 | | | 73.50 ± 13.78 | |  | 2.37 (-4.13, 8.87) | |
| SWM Between errors 4 boxes | 58 | 5.81 ± 4.57 | 5.54 ± 3.74 |  | 0.24 (-2.59, 3.08) | | | 157 | | | 5.71 ± 3.32 | | | 5.31 ± 3.59 | |  | 0.21 (-1.40, 1.82) | |
| SWM Between errors 6 boxes | 58 | 22.13 ± 6.34 | 23.31 ± 7.23 |  | 1.20 (-3.63, 6.03) | | | 156 | | | 24.48 ± 6.88 | | | 22.66 ± 5.83 | |  | -0.85 (-4.05, 2.36) | |
| SWM Between errors 8 boxes | 58 | 44.47 ± 6.93 | 45.38 ± 9.74 |  | -0.40 (-5.70, 4.90) | | | 153 | | | 44.90 ± 8.59 | | | 45.53 ± 7.77 | |  | 3.08 (-0.72, 6.87) | |
| SWM Strategy | 58 | 38.28 ± 2.98 | 37.19 ± 3.90 |  | 0.24 (-2.00, 2.49) | | | 153 | | | 38.21 ± 3.14 | | | 36.59 ± 3.05 | |  | -1.80 (-3.34, -0.26)^4^ | |

*CANTAB* Cambridge Neuropsychological Test Automated Battery, *SWM* Spatial Working Memory.

Values are adjusted mean differences (95% CI) from the reference group (Breast + bottle or bottle only).

^2^Values are adjusted mean differences (95% CI) from the reference group (Formula only).

^3^Adjusted models include the covariates: ethnicity (Chinese, Malay Indian), maternal education (non-tertiary, tertiary), birth weight category (SGA, AGA, LGA), 26-wk STAI-state scores (continuous), child’s sex, age during assessment (continuous).

^4^Values are P<0.05 compared to the reference group.

**Supplementary Table 6 Association between (a) the type of nursing and (b) milk nutrients and social emotional development assessments.**

| Social emotional development assessments |  | (a) Nursing | | | | | | | | | | (b) Nutrients | | | | | | | | | | | | | | | | | | | | | |  |
| --- | --- | --- | --- | --- | --- | --- | --- | --- | --- | --- | --- | --- | --- | --- | --- | --- | --- | --- | --- | --- | --- | --- | --- | --- | --- | --- | --- | --- | --- | --- | --- | --- | --- | --- |
|  | *N* | Unadjusted mean ± SD | | | |  | | | Adjusted mean differences (95% CI)^1,3^ | | | | | |  | | | Unadjusted mean ± SD | | | | | | | | |  | | Adjusted mean differences (95% CI)^2,3^ | | | | |  |
|  |  | Breast + bottle or bottle only | At breast only | | | |  | | | At breast only | |  | | *N* | | | | | Formula only | | | Breast milk + formula or breast milk only | | | |  | | | | | Breast milk + formula or breast milk only | | |  |
| Novel word learning | |  |  | |  | |  | | | |  |  | | | | |  | | | |  | | |  |  | | | | |  | | |  |  |
| 54 months |  |  |  | |  | |  | | | |  |  | | | | |  | | | |  | | |  |  | | | | |  | | |  |  |
| Test 1 (%) | 50 | 85.43 ± 11.22 | | 81.61 ± 17.32 | | |  | -8.89 (-22.37, 4.60) | | | | |  | | | 115 | | | | 74.46 ± 18.83 | | | 73.97 ± 24.69 | | | | |  | | | | -5.05 (-15.61, 5.50) | | |
| Test 2 (%) | 50 | 83.14 ± 16.29 | | 78.79 ± 15.88 | | |  | -3.00 (-16.50, 10.50) | | | | |  | | | 115 | | | | 76.63 ± 18.57 | | | 76.17 ± 26.85 | | | | |  | | | | -7.07 (-18.09, 3.96) | | |

^1^Values are adjusted mean differences (95% CI) from the reference group (Breast + bottle or bottle only).

^2^Values are adjusted mean differences (95% CI) from the reference group (Formula only).

^3^Adjusted models include the covariates: ethnicity (Chinese, Malay Indian), maternal education (non-tertiary, tertiary), birth weight category (SGA, AGA, LGA), 26-wk STAI-state scores (continuous), child’s sex and age at testing.

**Supplementary Table 7. F-statistic and P-values for the association between (a) the type of nursing and (b) milk nutrients with performance in memory assessments.**

|  | (a) Nursing | | | | |  | | | | (b) Nutrients | | | | | | | | | | |  |  |  |  |
| --- | --- | --- | --- | --- | --- | --- | --- | --- | --- | --- | --- | --- | --- | --- | --- | --- | --- | --- | --- | --- | --- | --- | --- | --- |
|  | Unadjusted model | |  | Adjusted model^2^ | | |  | Unadjusted model | | | | |  | | Adjusted model^2^ | | | | | | |  |  |  |
| Assessments of memory | F-statistic | P-value |  | F-statistic | P-value | |  | | F-statistic | | P-value | | |  | | F-statistic | | | P-value | | | | |  |
| Relational binding | |  |  |  |  | |  |  | | | |  |  | |  | |  | | | | | |  |  |
| 6 months |  |  |  |  |  | |  |  | | | |  |  | |  | |  | | | | | |  |  |
| Lag 0 trials (Time bins^1^) |  |  |  |  |  | |  |  | | | |  |  | |  | |  | | | | | |  |  |
| 1000-ms Bin 1 | 2.703 | 0.110 |  | 1.901 | 0.182 | |  | 6.156 | | | | 0.015 |  | | 2.121 | | | 0.149 | | | | | | |
| 1000-ms Bin 2 | 0.301 | 0.588 |  | 0.350 | 0.562 | |  | 0.444 | | | | 0.507 |  | | 0.096 | | | 0.757 | | | | | | |
| 1000-ms Bin 3 | 0.079 | 0.781 |  | 0.175 | 0.681 | |  | 0.661 | | | | 0.419 |  | | 2.074 | | | 0.155 | | | | | | |
| Lag 2 trials (Time bins^1^) |  |  |  |  |  | |  |  | | | |  |  | |  | | |  | | | | | | |
| 1000-ms Bin 1 | <0.001 | 0.982 |  | 0.281 | 0.602 | |  | 1.235 | | | | 0.270 |  | | 1.052 | | | 0.309 | | | | | | |
| 1000-ms Bin 2 | 0.038 | 0.847 |  | 0.416 | 0.528 | |  | 0.006 | | | | 0.938 |  | | 0.536 | | | 0.467 | | | | | | |
| 1000-ms Bin 3 | 1.581 | 0.222 |  | 6.715 | 0.022 | |  | 0.358 | | | | 0.552 |  | | 0.103 | | | 0.749 | | | | | | |
|  |  |  |  |  |  | |  |  | | | |  |  | |  | | |  | | | | | | |
| 41 months |  |  |  |  |  | |  |  | | | |  |  | |  | | |  | | | | | | |
| Accuracy in food block | 1.186 | 0.281 |  | 0.863 | 0.358 | |  | 0.158 | | | | 0.692 |  | | 0.572 | | | 0.451 | | | | | | |
| Accuracy in face block | 4.473 | 0.034 |  | 4.563 | 0.038 | |  | 0.760 | | | | 0.784 |  | | 0.098 | | | 0.320 | | | | | | |
| Combined food and face accuracy | 0.398 | 0.530 |  | 0.928 | 0.341 | |  | 0.290 | | | | 0.591 |  | | 1.626 | | | 0.205 | | | | | | |
| Inference memory accuracy | 0.606 | 0.440 |  | 1.181 | 0.283 | |  | 3.470 | | | | 0.065 |  | | 0.369 | | | 0.545 | | | | | | |
|  |  |  |  |  |  | |  |  | | | |  |  | |  | | |  | | | | | | |
| 54 months |  |  |  |  |  | |  |  | | | |  |  | |  | | |  | | | | | | |
| Lag 0 trials |  |  |  |  |  | |  |  | | | |  |  | |  | | |  | | | | | | |
| Accuracy | 1.938 | 0.170 |  | 0.070 | 0.792 | |  | 0.393 | | | | 0.532 |  | | 0.072 | | | 0.789 | | | | | | |
| % Looking to correct match | 0.216 | 0.644 |  | 1.986 | 0.167 | |  | 0.173 | | | | 0.678 |  | | 0.001 | | | 0.971 | | | | | | |
| Lag 2 trials |  |  |  |  |  | |  |  | | | |  |  | |  | | |  | |  | | | | |
| Accuracy | 1.080 | 0.304 |  | 0.544 | 0.465 | |  | 3.859 | | | | 0.052 |  | | 2.791 | | | 0.098 | | | | | | |
| % Looking to correct match | 0.984 | 0.326 |  | 4.983 | 0.031 | |  | 7.256 | | | | 0.008 |  | | 3.713 | | | 0.057 | | | | | | |
|  |  |  |  |  |  | |  |  | | | |  |  | |  | | |  | | | | | | |
| Habituation |  |  |  |  |  | |  |  | | | |  |  | |  | | |  | | | | | | |
| 6 months |  |  |  |  |  | |  |  | | | |  |  | |  | | |  | | | | | | |
| Proportion of time looking at novel stimuli | 0.890 | 0.352 |  | 0.250 | 0.621 | |  | 4.499 | | | | 0.036 |  | | 3.938 | | | 0.051 | | | | | | |
| Number of habituation trials | 0.003 | 0.958 |  | 0.527 | 0.475 | |  | 0.481 | | | | 0.490 |  | | 0.041 | | | 0.839 | | | | | | |
|  |  |  |  |  |  | |  |  | | | |  |  | |  | | |  | | | | | | |
| Deferred imitation |  |  |  |  |  | |  |  | | | |  |  | |  | | |  | | | | | | |
| 6 months |  |  |  |  |  | |  |  | | | |  |  | |  | | |  | | | | | | |
| Absolute score | 0.504 | 0.482 |  | 4.464 | 0.043 | |  | 1.073 | | | | 0.303 |  | | 0.746 | | | 0.789 | | | | | | |
|  |  |  |  |  |  | |  |  | | | |  |  | |  | | |  | | | | | | |
| 18 months |  |  |  |  |  | |  |  | | | |  |  | |  | | |  | | | | | | |
| Rattle task |  |  |  |  |  | |  |  | | | |  |  | |  | | |  | | | | | | |
| Absolute score | 0.976 | 0.337 |  | 1.233 | 0.309 | |  | <0.001 | | | | 0.984 |  | | 2.580 | | | 0.121 | | | | | | |
| Sequential score | 0.003 | 0.957 |  | 0.020 | 0.888 | |  | 0.237 | | | | 0.628 |  | | 2.564 | | | 0.114 | | | | | | |
|  |  |  |  |  |  | |  |  | | | |  |  | |  | | |  | | | | | | |
| 24 months |  |  |  |  |  | |  |  | | | |  |  | |  | | |  | | | | | | |
| Absolute score | 1.205 | 0.277 |  | 0.053 | 0.818 | |  | 1.464 | | | | 0.229 |  | | 0.417 | | | 0.520 | | | | | | |
| Sequential score | 3.778 | 0.057 |  | 1.454 | 0.235 | |  | 5.347 | | | | 0.022 |  | | 3.384 | | | 0.068 | | | | | | |
|  |  |  |  |  |  | |  |  | | | |  |  | |  | | |  | | | | | | |
| 41 months |  |  |  |  |  | |  |  | | | |  |  | |  | | |  | | | | | | |
| Absolute score for Dragonfly task | 0.452 | 0.504 |  | 0.337 | 0.565 | |  | 0.454 | | | | 0.502 |  | | 1.005 | | | 0.319 | | | | | | |
| Absolute score for Giraffe task | 0.002 | 0.962 |  | 0.670 | 0.798 | |  | 0.528 | | | | 0.469 |  | | 0.093 | | | 0.761 | | | | | | |
| Absolute score for Bird task | 3.819 | 0.056 |  | 3.246 | 0.079 | |  | 1.031 | | | | 0.312 |  | | 0.061 | | | 0.805 | | | | | | |

^1^Time bins are defined in 1000-ms blocks after the pictures appear on the screen.

^2^Values are adjusted for ethnicity (Chinese, Malay Indian), maternal education (non-tertiary, tertiary), birth weight category (SGA, AGA, LGA), 26-wk STAI-state scores (continuous), child’s sex, age during assessment (continuous).

**Supplementary Table 8. F-statistic and P-values for the association between (a) the type of nursing and (b) milk nutrients with performance in testing batteries.**

|  | (a) Nursing | | | | |  | | | | (b) Nutrients | | | | | | | | | | |  |  |  |  |  |
| --- | --- | --- | --- | --- | --- | --- | --- | --- | --- | --- | --- | --- | --- | --- | --- | --- | --- | --- | --- | --- | --- | --- | --- | --- | --- |
|  | Unadjusted model | |  | Adjusted model | | |  | Unadjusted model | | | | |  | | Adjusted model | | | | | | |  |  |  |  |
| Testing batteries | F-statistic | P-value |  | F-statistic | P-value | |  | | F-statistic | | P-value | | |  | | F-statistic | | | P-value |  |  |  |  |  |  |
| BSID-III | |  |  |  |  | |  |  | | | |  |  | |  | |  | | | | | |  |  |  |
| 24 months |  |  |  |  |  | |  |  | | | |  |  | |  | |  | | | | | |  |  |  |
| Cognition | 3.248 | 0.077 |  | 2.402 | 0.127 | |  | 9.098 | | | | 0.003 |  | | 6.668 | | 0.011 | | | | | |  |  |  |
| Receptive language | 0.209 | 0.649 |  | 0.077 | 0.783 | |  | 6.629 | | | | 0.011 |  | | 0.647 | | 0.423 | | | | | | |  |  |
| Expressive language | 0.219 | 0.641 |  | 0.006 | 0.940 | |  | 5.799 | | | | 0.017 |  | | 1.181 | | 0.279 | | | | | | |  |  |
| Fine motor | 2.249 | 0.139 |  | 3.113 | 0.084 | |  | 2.928 | | | | 0.089 |  | | 1.118 | | 0.292 | | | | | | |  |  |
| Gross motor | 0.011 | 0.915 |  | 0.115 | 0.736 | |  | 5.601 | | | | 0.019 |  | | 4.398 | | 0.038 | | | | | | |  |  |
|  |  |  |  |  |  | |  |  | | | |  |  | |  | |  | | | | | | |  |  |
| KBIT-2  54 months |  |  |  |  |  | |  |  | | | |  |  | |  | |  | | | | | | |  |  |
| Verbal | 0.179 | 0.674 |  | 0.072 | 0.790 | |  | 16.366 | | | | <0.001 |  | | 4.071 | | 0.046 | | | | | | |  |  |
| Nonverbal | 2.659 | 0.108 |  | 1.107 | 0.298 | |  | 5.033 | | | | 0.026 |  | | 3.295 | | 0.072 | | | | | | |  |  |
| IQ | 1.643 | 0.205 |  | 0.632 | 0.430 | |  | 15.314 | | | | <0.001 |  | | 5.509 | | 0.020 | | | | | | |  |  |
|  |  |  |  |  |  | |  |  | | | |  |  | |  | | |  | | | | | | |  |
| School readiness test |  |  |  |  |  | |  |  | | | |  |  | |  | | |  | | | | | | |  |
| 48 months |  |  |  |  |  | |  |  | | | |  |  | |  | | |  | | | | | | |  |
| Lollipop score^1^ | 4.144 | 0.044 |  | 3.601 | 0.061 | |  | 16.532 | | | | <0.001 |  | | 0.170 | | | 0.681 | | | | | | |  |
| NK test scores^1^ |  |  |  |  |  | |  |  | | | |  |  | |  | | |  | | | | | | |  |
| Test 0 | 3.328 | 0.071 |  | 1.881 | 0.174 | |  | 9.011 | | | | 0.003 |  | | 0.629 | | | 0.429 | | | | | | |  |
| Test 1 | 0.326 | 0.569 |  | 0.133 | 0.716 | |  | 10.232 | | | | 0.002 |  | | 0.020 | | | 0.887 | | | | | | |  |
| Total | 0.919 | 0.340 |  | 0.433 | 0.512 | |  | 13.540 | | | | <0.001 |  | | 0.199 | | | 0.656 | | | | | | |  |
| PPVT score^1^ | 5.700 | 0.019 |  | 4.396 | 0.039 | |  | 18.132 | | | | <0.001 |  | | 1.903 | | | 0.169 | | | | | | |  |
| ROST score^1^ | 2.200 | 0.141 |  | 1.020 | 0.315 | |  | 0.002 | | | | 0.968 |  | | <0.001 | | | 0.999 | | | | | | |  |
| VCR score^3^ | 0.520 | 0.473 |  | 2.096 | 0.152 | |  | 0.206 | | | | 0.650 |  | | 0.391 | | | 0.532 | | | | | | | |
| CTOPP2 scores^2^ |  |  |  |  |  | |  |  | | | |  |  | |  | | |  | | | | | | |  |
| Elision | 0.576 | 0.450 |  | 0.042 | 0.839 | |  | 4.801 | | | | 0.029 |  | | 0.297 | | | 0.586 | | | | | | |  |
| Blending Words | 0.627 | 0.430 |  | <0.001 | 0.994 | |  | 4.771 | | | | 0.030 |  | | 0.002 | | | 0.964 | | | | | | |  |
| Sound matching | 0.510 | 0.477 |  | 0.763 | 0.395 | |  | 3.551 | | | | 0.061 |  | | 0.585 | | | 0.445 | | | | | | |  |
| Phonological awareness | 0.705 | 0.403 |  | 0.001 | 0.973 | |  | 6.380 | | | | 0.012 |  | | 0.261 | | | 0.610 | | | | | | |  |
| Panamath^1^ |  |  |  |  |  | |  |  | | | |  |  | |  | | |  | | | | | | |  |
| % correct trials | 0.004 | 0.949 |  | 3.915 | 0.056 | |  | 0.015 | | | | 0.902 |  | | 0.128 | | | 0.722 | | | | | | |  |
| Weber fraction | 0.204 | 0.653 |  | 6.861 | 0.013 | |  | 0.530 | | | | 0.469 |  | | 0.078 | | | 0.781 | | | | | | |  |

*NK* Number Knowledge, *PPVT* Peabody Picture Vocabulary Test, *ROST* Random Object Span Test, *VCR* Visually Cued Recall Test, *CTOPP2* Comprehensive Test of Phonological Processing-2.

^1^Adjusted models include the covariates: ethnicity (Chinese, Malay Indian), maternal education (non-tertiary, tertiary), birth weight category (SGA, AGA, LGA), 26-wk STAI-state scores (continuous), child’s sex and age at testing.

^2^Adjusted models include the covariates: ethnicity (Chinese, Malay Indian), maternal education (non-tertiary, tertiary), birth weight category (SGA, AGA, LGA), 26-wk STAI-state scores (continuous) and child’s sex.

**Supplementary Table 9. F-statistic and P-values for the association between (a) the type of nursing and (b) milk nutrients with assessments of executive functioning and self-regulation.**

|  | (a) Nursing | | | | |  | | | | (b) Nutrients | | | | | | | | | |  |  |  |  |
| --- | --- | --- | --- | --- | --- | --- | --- | --- | --- | --- | --- | --- | --- | --- | --- | --- | --- | --- | --- | --- | --- | --- | --- |
|  | Unadjusted model | |  | Adjusted model^1^ | | |  | Unadjusted model | | | | |  | | Adjusted model^1^ | | | | | |  |  |  |
| Assessments of executive functioning and self-regulation | F-statistic | P-value |  | F-statistic | P-value | |  | | F-statistic | | P-value | | |  | | F-statistic | | P-value |  |  |  |  |  |
| Dimensional change card sorting task | |  |  |  |  | |  |  | | | |  |  | |  | |  | | | | |  |  |
| 41 months |  |  |  |  |  | |  |  | | | |  |  | |  | |  | | | | |  |  |
| Passed Emotion task | NA | 0.814 |  | NA | 0.619 | |  | NA | | | | 0.023 |  | | NA | | 0.546 | | | | |  |  |
| Passed Food task | NA | 0.877 |  | NA | 0.390 | |  | NA | | | | 0.057 |  | | NA | | 0.918 | | | | | |  |
|  |  |  |  |  |  | |  |  | | | |  |  | |  | |  | | | | | |  |
| 54 months |  |  |  |  |  | |  |  | | | |  |  | |  | |  | | | | | |  |
| Performance in emotion task | NA | 0.181 |  | NA | 0.222 | |  | NA | | | | 0.860 |  | | NA | | 0.861 | | | | | |  |
| Performance in orientation task | NA | 0.338 |  | NA | 0.862 | |  | NA | | | | 0.784 |  | | NA | | 0.519 | | | | | | |
|  |  |  |  |  |  | |  |  | | | |  |  | |  | |  | | | | | | |
| Sticker and Snack delay |  |  |  |  |  | |  |  | | | |  |  | |  | |  | | | | | | |
| 41 months |  |  |  |  |  | |  |  | | | |  |  | |  | |  | | | | | | |
| Snack Delay score | 0.352 | 0.555 |  | 0.395 | 0.532 | |  | 0.300 | | | | 0.585 |  | | 0.568 | | 0.453 | | | | | | |
| Sticker Delay score | 1.639 | 0.206 |  | 3.103 | 0.085 | |  | 0.818 | | | | 0.368 |  | | 0.052 | | 0.819 | | | | | | |
| Snack & Sticker Delay score | 1.688 | 0.199 |  | 3.818 | 0.057 | |  | 0.026 | | | | 0.872 |  | | 0.115 | | 0.735 | | | | | | |

^1^Adjusted models include the covariates: ethnicity (Chinese, Malay Indian), maternal education (non-tertiary, tertiary), birth weight category (SGA, AGA, LGA), 26-wk STAI-state scores (continuous), child’s sex and age at testing.

**Supplementary Table 10. F-statistic and P-values for the association between (a) the type of nursing and (b) milk nutrients with assessments of attention/ pre-attention and working memory.**

|  | (a) Nursing | | | | |  | | | | (b) Nutrients | | | | | | | | | |  |  |  |  |
| --- | --- | --- | --- | --- | --- | --- | --- | --- | --- | --- | --- | --- | --- | --- | --- | --- | --- | --- | --- | --- | --- | --- | --- |
|  | Unadjusted model | |  | Adjusted model^1^ | | |  | Unadjusted model | | | | |  | | Adjusted model^1^ | | | | | |  |  |  |
| Assessments of attention/ pre-attention and working memory | F-statistic | P-value |  | F-statistic | P-value | |  | | F-statistic | | P-value | | |  | | F-statistic | | P-value |  |  |  |  |  |
| Visual expectation | |  |  |  |  | |  |  | | | |  |  | |  | |  | | | | |  |  |
| 6 months |  |  |  |  |  | |  |  | | | |  |  | |  | |  | | | | |  |  |
| Reaction time at experimental phase (ms) | 0.195 | 0.662 |  | 0.055 | 0.817 | |  | 1.682 | | | | 0.198 |  | | 0.899 | | 0.346 | | | | |  |  |
| Proportion of time looking at correct location (%) | 0.004 | 0.949 |  | 0.020 | 0.889 | |  | 0.508 | | | | 0.478 |  | | 0.928 | | 0.338 | | | | | |  |
|  |  |  |  |  |  | |  |  | | | |  |  | |  | |  | | | | | |  |
| 18 months |  |  |  |  |  | |  |  | | | |  |  | |  | |  | | | | | |  |
| Reaction time at experimental phase (ms) | 0.546 | 0.465 |  | 0.297 | 0.590 | |  | 0.558 | | | | 0.457 |  | | 0.211 | | 0.647 | | | | | |  |
| Proportion of time looking at correct location (%) | 1.229 | 0.275 |  | 1.789 | 0.192 | |  | 0.369 | | | | 0.545 |  | | 0.294 | | 0.726 | | | | | | |
|  |  |  |  |  |  | |  |  | | | |  |  | |  | |  | | | | | | |
| CANTAB Spatial working memory |  |  |  |  |  | |  |  | | | |  |  | |  | |  | | | | | | |
| 54 months |  |  |  |  |  | |  |  | | | |  |  | |  | |  | | | | | | |
| SWM Between errors | 0.245 | 0.623 |  | 0.050 | 0.824 | |  | 0.284 | | | | 0.595 |  | | 0.519 | | 0.472 | | | | | | |
| SWM Between errors 4 boxes | 0.060 | 0.807 |  | 0.030 | 0.864 | |  | 0.357 | | | | 0.551 |  | | 0.067 | | 0.796 | | | | | | |
| SWM Between errors 6 boxes | 0.440 | 0.510 |  | 0.250 | 0.619 | |  | 1.903 | | | | 0.170 |  | | 0.273 | | 0.602 | | | | | | |
| SWM Between errors 8 boxes | 0.174 | 0.678 |  | 0.023 | 0.880 | |  | 0.142 | | | | 0.707 |  | | 2.576 | | 0.111 | | | | | | |
| SWM Strategy | 1.455 | 0.233 |  | 0.048 | 0.828 | |  | 6.764 | | | | 0.010 |  | | 5.329 | | 0.023 | | | | | | |

*CANTAB* Cambridge Neuropsychological Test Automated Battery, *SWM* Spatial Working Memory.

^1^Adjusted models include the covariates: ethnicity (Chinese, Malay Indian), maternal education (non-tertiary, tertiary), birth weight category (SGA, AGA, LGA), 26-wk STAI-state scores (continuous), child’s sex, age during assessment (continuous).

Supplementary Table 11. F-statistic and P-values for the association between (a) the type of nursing and (b) milk nutrients with social emotional development assessments.

|  | (a) Nursing | | | | |  | | | | (b) Nutrients | | | | | | | | |  |  |  |
| --- | --- | --- | --- | --- | --- | --- | --- | --- | --- | --- | --- | --- | --- | --- | --- | --- | --- | --- | --- | --- | --- |
|  | Unadjusted model | |  | Adjusted model^1^ | | |  | Unadjusted model | | | | |  | | Adjusted model^1^ | | | | |  |  |
| Social emotional development assessments | F-statistic | P-value |  | F-statistic | P-value | |  | | F-statistic | | P-value | | |  | | F-statistic | | P-value |  |  |  |
| Novel word learning | |  |  |  |  | |  |  | | | |  |  | |  | |  | | | |  |
| 54 months |  |  |  |  |  | |  |  | | | |  |  | |  | |  | | | |  |
| Test 1 (%) | 2.517 | 0.119 |  | 1.779 | 0.190 | |  | 0.012 | | | | 0.914 |  | | 0.903 | | 0.344 | | | |  |
| Test 2 (%) | 0.917 | 0.343 |  | 0.203 | 0.655 | |  | 0.010 | | | | 0.921 |  | | 1.619 | | 0.206 | | | | |

^1^Adjusted models include the covariates: ethnicity (Chinese, Malay Indian), maternal education (non-tertiary, tertiary), birth weight category (SGA, AGA, LGA), 26-wk STAI-state scores (continuous), child’s sex and age at testing.

**Supplementary Methods: Detailed description of the cognitive test methodologies from 6 to 54 months.**

The neurocognitive assessment window periods for the different time points were: 6 month (M) visit: 6M ± 2 wk; 18M visit: 17–19 months; 24M visit: 23–25 months; 41M visit: 40-42 months; 48M visit: 48-50 months; and 54M visit: 54-56 months.

**Memory Tasks**

Habituation: 6 months

Habituation measures infant memory and is predictive of childhood cognitive functioning [1]. Specific details of our experiment have been reported elsewhere [2]. Briefly, each trial length began after the infant had looked at the stimulus (either a picture of a bear or a wolf) and ended when the infant looked away, both for at least for one second. The infant was considered to have habituated when the sum of the infant’s look time in three consecutive trials became less than half the criterion, which was set as the sum of the three consecutive longest trial durations.

The habituation program recorded the number of trials it took for an infant to habituate as well as the longest length of time an infant looked during any one trial. After the infant has habituated, a novel stimulus was presented on one side of the monitor and the habituated stimulus was presented on the other. After 10 seconds, the images were presented on opposite sides for another 10 seconds. The infant’s looking behavior was video recorded via a camera discreetly positioned above the monitor and was assessed later. Coders were blinded as to which image appeared on the left and right of the screen and judged where the infant was looking without knowing at which object the infant was looking. The scores were then matched against a master file from which we determined the average proportion of time the baby looked at a novel item, and considered the duration the infant looked away from the screen entirely.

Deferred Imitation: 6, 18, 24 and 41 months

Deferred imitation is a recognized way of assessing declarative memory in non-verbal groups like infants [3]. The deferred imitation tasks used at 6 months (puppet paradigm) and 18 months (rattle paradigm) were adapted from Barr et al. [4] The (slide) task used at 24 months old was modified from Kolling et al. [5] Specific details of our experiments have been previously reported [2]. Each deferred imitation experiment started with a baseline phase where the infant could interact with the toys/items to determine whether he/she could spontaneously produce any of the target actions. After the baseline phase, the experimenter illustrated the target actions with the items provided. A test occurred after a 2-3 hour delay following the training phase. The infant was presented with the same items in the same context to determine whether they displayed imitation of the trained actions. The entire procedure was video recorded and scored for target behaviors. Absolute and sequential scores were given based on number of target actions and correct sequences achieved.

For deferred imitation at 41 months, three video-recorded tasks (i.e., “Bird,” “Dragonfly,” and “Giraffe”) were adapted from Kolling et al. [6]: During the baseline phase of each task children were given a set of novel objects. Next, an experimenter demonstrated how the objects could be used in conjunction with one another. The tasks differed in the degree to which the actions were goal oriented (e.g., items used to make an animal shape or not), required performance in a certain sequence, and in the number of demonstrated actions. Two to three hours after demonstration the children were again allowed to interact with the items. The video records were scored offline to determine the number and sequence of produced target actions at base-line and following learning plus delay.

Relational Binding (RB): 6, 41 and 54 months

The memory paradigm [7] for the RB task at 6 months is a modification of that used by Richmond et al. [8] Specific details of our experiment have been reported elsewhere [2]. Briefly, infants were shown blocks of trials involving scenes and pictures of toys. Each block consists of three study trials and one test trial. Each study trial consists of an audiovisual scene with a picture of a toy superimposed. During the test trial, the infants were shown one of the three audiovisual scenes, superimposed with all three pictures of toys viewed previously. The proportion of time the child spent looking at the various pictures or background was captured by the eye tracker. Relational memory is inferred when the child preferentially looks at the correct match (e.g., looks longer at the correctly matched toy-and-background pair). We included Lag 0 (matched pair of scene/toy appeared in the study trial immediately before the test trial) and Lag 2 (matched pair of scene/toy appeared two study trials before the test trial) trials. Lag 2 trials involved some delay and interference from other stimuli and hence are of a greater difficulty level than Lag 0 trials.

The computerized RB tasks conducted at 41 month and 54 months are conceptually similar to the task conducted at 6 months, though, structurally the 41-month task differed [8]. At 41 months, attractive food and emotional faces were used as stimuli. Children were shown blocks of trials involving animals and faces/food. Each block consisted of 10 study trials, each of which was repeated a second time, and 5 test trials. Each study trial consisted of an animal displayed together with 2 pictures of faces/food. During the test trials, children were shown 1 of the 10 animals, with 2 different faces/food viewed previously; they had to identify the face/food that was previously depicted alongside the animal. In the inference memory block, children were shown pictures of food and asked to identify the person that had been presented alongside the animal that was shown with the food (i.e., utilizing information they had learned in both the face and food blocks). Both socioemotional (i.e., faces) and non-socioemotional (i.e., food) stimuli were included to allow for the differential examination of past experience and concurrent emotional/motivational state upon memory. Past work suggests that arousal during encoding can selectively influence memory for affective, but not neutral, information [9].

Similar to the 6 month time point, and as adapted from Richmond and Nelson [8] as well as Hannula et al. [10], at 54 months, the relational memory task was administered via a 17” square eye-tracker, E-Prime version 2.0 with TOBII extensions. Each of 15 trials began with children seeing three unique toys superimposed upon three unique audiovisual scenes. Then, at the test phase of each trial, children were shown one of the three trial’s audiovisual scenes with all three of that trial’s toys superimposed upon it and asked to indicate which of the three toys had previously been paired with the audiovisual scenes. The audiovisual scene displayed at test was always either the first scene to have been presented or the last scene to have been presented before test; accordingly, then, there was either a lag of 2 audiovisual scenes between the initial presentation of the audiovisual scene and the test presentation of the audiovisual scene (i.e., “Lag 2”) or no intervening slides between the presentation of an audiovisual scene and the second presentation of the same audiovisual scene (i.e., “Lag 0”).

**Executive Functioning and Self-regulation**

Dimensional Card Change Sort (DCCS): 41 and 54 months

The DCCS is a card-sorting task that examines the child’s ability to learn and apply new rules while refraining from using old rules [11]. For example, children may be asked to sort bivalent pictures according to color and then subsequently by shape. At 41 months, children were given two versions of the DCCS. One with colored pictures of food (i.e., ice-cream and cake) and one with colored pictures of emotional icons (i.e., a happy or an angry cartoon face). Children did not repeat the two versions back-to-back, and the order in which they received them was counterbalanced. For both versions, children were asked to manually sort cards into boxes according to a given rule and this rule remained consistent throughout a block of 6 trials. For example, in the “food” condition, children were given a set of cards depicting, e.g., blue ice cream cones or red pieces of cake, and asked to first sort 6 cards into one of two boxes labelled with e.g., a red ice cream cone and a blue piece of cake, according to the color of the cards. When the rule for a block was “sort by color” the blue ice cream-cone would correctly be put into a box labelled with a “blue piece of cake.” Next, they were asked to sort 6 cards by shape, and in this condition the “blue ice cream cone” would correctly be placed into the box labelled with the red ice-cream cone.

At 54 months, the paradigm was administered on a computer with press pad, using E-Prime version 2.0. Similar to what occurred at 41 months, children were first asked to sort bivalent pictures according to one rule for an entire block of 5 trials, and then according to a different rule for an entire block of 5 trials. Also similar to 41 months, the DCCS was administered twice. In both conditions one of the rules included “sort by color.” However, depending on the condition, the other rule was either to sort by spatial orientation or by emotional expression. That is, in one condition (not emotional) children saw neutral colored faces that were either upside down or right side up. In the other condition (emotional) they saw colored happy or angry faces.

At 54 months an additional block was added for children who passed the simpler conditions. In this last “mixed trials” block consisting of 30 trials, an instruction word and sound cue preceded each trial and indicated the rule for sorting the test stimuli in that trial (i.e. either by “color” or by “face”).

Snack and Sticker Delay: 41 months

The snack delay and Sticker Delay tasks were used to measure delayed gratification, which is a function of effortful control. Effortful control refers to the ability to suppress a dominant response in order to perform a subdominant response [12]. The child was first given a snack/sticker to eat/paste. The experimenter then placed one snack/sticker under a transparent plastic cup and instructed the child to wait for the experimenter to ring a bell before retrieving the snack/sticker. Two practice trials were followed by four separate test trials with delay intervals of 10s, 20s, 30s, and then 15s. The test was video-recorded and scored according to Kochanska and colleagues [13].

**Attention/pre-attention and Working Memory**

Visual expectation: 6 and 18 months

Visual expectation paradigms assess infant attention, primarily attention orientation [14,15]. Performance in such paradigms relates to other cognitive processes, including recognition memory [16] and novelty detection [17]. Specific details of our experiment have been reported elsewhere [2]. Briefly, our visual expectation paradigm began with infants viewing 18 infant-appropriate movie clips presented in random locations on a screen. Next, infants took part in the experimental portion, in which they watched similar clips that were presented in locations on the screen that varied according to a specified pattern (e.g., left-left-right). Stimuli were presented for 700 ms (6 months) or 2500 ms (18 months) with an inter-stimulus interval of 1000 ms. Variables of interest used in this analysis were computed, including (1) basic eye movement - the time taken during the random control portion of the experiment for an individual to shift his/her gaze from a different location of the screen to the one where the stimuli eventually appeared; (2) reaction time –the average time taken for an individual to shift his/her gaze during the experimental portion of the task; (3) average proportion of time spent looking at stimuli presented; (4) proportion of trials where infants correctly anticipated (i.e. looked at where the stimuli were expected to appear next); (5) average duration of anticipation when it occurred; and (6) average proportion of time spent looking at the correct location in advance of the stimulus appearing on the screen.

CANTAB Spatial Working Memory: 54 months

The Spatial Working Memory (SWM) test from the Cambridge Neuropsychological Test of Automated Battery (CANTAB) was administered on a computer fitted with a touch screen to measure the child’s ability to maintain and update spatial information using working memory [18]. The test began with several boxes shown on the screen. By process of elimination, the child was asked to find one ‘token’ in each of a number of boxes. The difficulty of the test was increased by gradually increasing the number of boxes, from 4 to a maximum of 8. The error score was derived from revisiting boxes that had already been found to be empty or had already been found to contain a token. The extent to which a pre-determined sequence was used is an indication of the child’s strategic thinking ability.

**Social-Emotional Development**

Novel Word Learning Paradigm: 54 months

This paradigm was adapted from Gliga et al. [19], with details of the experiment described elsewhere [20]. Briefly, the stimuli were presented on a video screen in English by the same adult. During the task, children were shown pairs of objects: a novel static object (non-changing and non-moving) and a novel noticeable distractor (changed color or had moving parts); the correct referent of a novel word is indicated by following the gaze of the adult. Eight video clips were shown to the child, each consisting of a familiarization phase (with 2 intervals: baseline and teaching) and a test phase consisting of 2 types of tests: a reference test and a mutual exclusivity test.

**Testing batteries**

Bayley Scales of Infant and Toddler Development, 3^rd^ edition (BSID-III): 24 months

The BSID-III is a standardized test that assesses development across a number of domains for children 1–42 months of age. It provides five US age-norm-referenced subscale scores for cognition, expressive language, receptive language, fine motor, and gross motor [21]. The BSID-III was administered by research coordinators trained by clinicians from the KK Women’s and Children’s Hospital.

School Readiness Test: 48 months

Children were assessed for school readiness using various tests at 48 months. The Lollipop Test contains 4 sections: (i) identification of colours and shapes and description of shapes; (ii) description of images, positions and spatial identification; (iii) identification of numbers and calculations; and (iv) identification of letters and handwriting [22]. The Number Knowledge Test (NKT) measures basic knowledge and understanding of numerical concepts and was administered in two levels: Level 0 assesses the child's ability to quantify objects that can be seen or touched; Level 1 relies on the child’s ability to perform mental calculation with limited visual aids [23]. The Random Object Span Test (ROST) measures working memory, and can be seen as an age-appropriate version of the Self-Ordered Pointing (SOP) task [24]. The child is first shown an array with two pictures (level 2) and is asked to select one of them. Then a second array of the same two pictures is presented again but in different locations. The child is asked to select another picture. A new level 2 array with new pictures is then presented. Then a level with 3 arrays is introduced and so on. There are two different trials per level with arrays of gradually increasing number of pictures. The Visually Cued Recall task (VCR) assesses working memory, using a combination of a pictorial memory span task and a delayed response task [25]. The child was asked to remember a gradually increasing span of items shown on a card. The Peabody Picture Vocabulary Test, 4th edition (PPVT-4) was used to assess receptive vocabulary [26]. The child was asked to select one of four pictures that best illustrates the meaning of the given word. Children’s phonological awareness (a prerequisite to reading fluency) was assessed with the Comprehensive Test of Phonological Processing, 2nd edition (CTOPP-2)[27]. The scores for PPVT and CTOPP-2 were age-standardised, based on a nationally representative sample of U.S. children. Panamath measures the child’s number sense and number system aptitude by assessing his or her ability to accurately decide whether there were more blue dots or yellow dots in a brief flash [28,29]. All tests of school readiness were administered in English.

Kaufman Brief Intelligence Test, 2nd edition (KBIT-2): 54 months

The KBIT-2 measures children’s verbal and nonverbal abilities [30]. For the verbal scale, the child was asked to provide one-word responses or point to one of 5 or 6 pictures that illustrated the meaning of the word or the answer to the question asked. The nonverbal scale (matrices) measures the understanding of relations among either concrete stimuli (pictures of objects or people) or abstract stimuli (designs or symbols). Each child obtained a standardized score with a mean of 100, with a SD of 15, for overall IQ Composite Score, as well as a Verbal Score and a Nonverbal Score.

**References**

1. Bornstein MH, Hahn CS, Bell C et al (2006) Stability in cognition across early childhood. A developmental cascade. Psychol Sci 17:151-158. http://dx.doi.org/10.1111/j.1467-9280.2006.01678.x

2. Cai S, Pang WW, Low YL et al (2015) Infant feeding effects on early neurocognitive development in Asian children. Am J Clin Nutr 101:326-336. http://dx.doi.org/10.3945/ajcn.114.095414

3. Meltzoff AN (1988) Infant imitation and memory: nine-month-olds in immediate and deferred tests. Child Dev 59:217-225

4. Barr R, Hayne H (1999) Developmental changes in imitation from television during infancy. Child Dev 70:1067-1081

5. Kolling T, Goertz C, Stefanie F, Knopf M (2010) Memory development throughout the second year: overall developmental pattern, individual differences, and developmental trajectories. Infant Behav Dev 33:159-167. http://dx.doi.org/10.1016/j.infbeh.2009.12.007

6. Kolling T, Knopf M (2015) Measuring declarative memory from infancy to childhood: The Frankfurt Imitation Tests for infants and children aged 12-36 months. European Journal of Developmental Psychology 12:359-376

7. Hannula DE, Tranel D, Cohen NJ (2006) The long and the short of it: relational memory impairments in amnesia, even at short lags. J Neurosci 26:8352-8359. http://dx.doi.org/10.1523/JNEUROSCI.5222-05.2006

8. Richmond J, Nelson CA (2009) Relational memory during infancy: evidence from eye tracking. Dev Sci 12:549-556. <http://dx.doi.org/10.1111/j.1467-7687.2009.00795.x>

9. Cahill, L., Gorski, L., & Le, K. (2003) Enhanced human memory consolidation with post-learning stress: Interaction with the degree of arousal at encoding. Learning & Memory 10:270–274.

10. Hannula DE, Ryan JD, Tranel D, Cohen NJ (2007) Rapid onset relational memory effects are evident in eye movement behavior, but not in hippocampal amnesia. J Cogn Neurosci 19:1690-1705. http://dx.doi.org/10.1162/jocn.2007.19.10.1690

11. Zelazo PD (2006) The Dimensional Change Card Sort (DCCS): a method of assessing executive function in children. Nat Protoc 1:297-301. http://dx.doi.org/10.1038/nprot.2006.46

12. Kochanska G, Murray KT, Harlan ET (2000) Effortful control in early childhood: Continuity and change, antecedents, and implications for social development. Developmental Psychology 36:220-232. http://dx.doi.org/10.1037/0012-1649.36.2.220

13. Kochanska G, Murray K, Jacques TY et al (1996) Inhibitory control in young children and its role in emerging internalization. Child Development 67:490-507. http://dx.doi.org/10.2307/1131828

14. Posner MI, Rothbart MK, Sheese BE, Voelker P (2012) Control networks and neuromodulators of early development. Dev Psychol 48:827-835. http://dx.doi.org/10.1037/a0025530

15. Rothbart MK, Sheese BE, Rueda MR, Posner MI (2011) Developing Mechanisms of Self-Regulation in Early Life. Emot Rev 3:207-213. http://dx.doi.org/10.1177/1754073910387943

16. Aguirre GK, Zarahn E, D'Esposito M (1998) An area within human ventral cortex sensitive to "building" stimuli: evidence and implications. Neuron 21:373-383

17. Markowska AL, Olton DS, Murray EA, Gaffan D (1989) A comparative analysis of the role of fornix and cingulate cortex in memory: rats. Exp Brain Res 74:187-201

18. Luciana M, Nelson CA (2002) Assessment of neuropsychological function through use of the Cambridge Neuropsychological Testing Automated Battery: Performance in 4- to 12-year-old children. Developmental Neuropsychology 22:595-624. http://dx.doi.org/10.1207/S15326942DN2203_3

19. Gliga T, Elsabbagh M, Hudry K et al (2012) Gaze following, gaze reading, and word learning in children at risk for autism. Child Development 83:926-938

20. Yow WQ, Li X, Lam S et al (2017) A bilingual advantage in 54-month-olds' use of referential cues in fast mapping. Dev Sci 20. http://dx.doi.org/10.1111/desc.12482

21. Bayley N (2006) Bayley Scales of Infant and Toddler Development–Third Edition: Administration Manual. In. Harcourt Assessment San Antonio: TX,

22. Chew AL (1981) The Lollipop Test, a Diagnostic Screening Test of School Readiness. Humanics,

23. Okamoto Y, Case R (1996) The role of central conceptual structures in the development of children's thought: II. Exploring the microstructure of children's central conceptual structures in the domain of number. Monographs of the Society for Research in Child Development 61:27-58. http://dx.doi.org/10.1111/j.1540-5834.1996.tb00536.x

24. Milner B, Petrides M (1984) Behavioural effects of frontal-lobe lesions in man. Trends in Neurosciences 7:403-407. http://dx.doi.org/10.1016/S0166-2236(84)80143-5

25. Zelazo PD, Jacques S, Burack JA, Frye D (2002) The relation between theory of mind and rule use: Evidence from persons with autism-spectrum disorders. Infant and Child Development 11:171-195. http://dx.doi.org/10.1002/icd.304

26. Dunn M, Dunn LM (1981) Peabody Picture Vocabulary Test - Revised. American Guidance Service, Circle Pines, MN

27. Wagner RK, Torgesen JK, Rashotte CA, Pearson NA (2013) Comprehensive test of phonological processing - 2nd ed. (CTOPP-2). Pro-ed, Austin, TX

28. Libertus ME, Feigenson L, Halberda J (2013) Is Approximate Number Precision a Stable Predictor of Math Ability? Learn Individ Differ 25:126-133. http://dx.doi.org/10.1016/j.lindif.2013.02.001

29. Halberda J, Mazzocco MM, Feigenson L (2008) Individual differences in non-verbal number acuity correlate with maths achievement. Nature 455:665-668. http://dx.doi.org/10.1038/nature07246

30. Kaufman AS, Kaufman NL (2004) Kaufman brief intelligence test. Wiley Online Library,
